# Configuration Investigation, Structure Design and Deployment Dynamics of Rigid-Reflector Spaceborne Antenna with Deviation-Angle Panel

**DOI:** 10.3390/s24020385

**Published:** 2024-01-08

**Authors:** Guodong Tan, Kaiqi Liu, Xuechao Duan, Qunbiao Wang, Dan Zhang, Dongwu Yang, Dingchao Niu

**Affiliations:** 1 Key Laboratory of Electronic Equipment Structure Design, Ministry of Education, Xidian University, Xi’an 710071, China; hbkaqsd@163.com (G.T.); kqliu@stu.xidian.edu.cn (K.L.); qbwang@stu.xidian.edu.cn (Q.W.); ydw_1978@126.com (D.Y.); niu_dc@163.com (D.N.); 2 Department of Mechanical Engineering, The Hong Kong Polytechnic University, Hong Kong, China; dan.zhang@polyu.edu.hk

**Keywords:** rigid-reflector spaceborne antenna, deviation-angle panel, stowing efficiency, Euler’s rotation theorem, parameter identification, deployment dynamics

## Abstract

Rigid-reflector spaceborne antennas (RRSAs) are well-suited for high-frequency application scenarios due to their high surface accuracy. However, the low stowing efficiency of RRSAs limits the aperture diameters and further deteriorates the electromagnetic (EM) performances in terms of gain, resolution and sensitivity. After conducting systematic feature analysis with respect to several typical RRSAs, we propose a novel type of RRSA to solve the aforementioned problems. Inspired by the pose adjustment process for a higher stowing efficiency of traditional RRSAs, we also propose a new segmentation scheme of a reflective surface consisting of a deviation-angle panel that facilitates a higher stowing efficiency. Based on this scheme, its corresponding folded configuration is implemented by combining Euler’s rotation theorem and the idea of parameter identification. In addition, we also compare the stowing efficiency of different schemes to verify the high stowing efficiency of the configuration. Finally, we perform mechanism/structure design and deployment dynamics to demonstrate that the antenna can be successfully deployed and exhibits excellent deployment quality. The results suggest that the proposed antenna possesses higher stowing efficiency than that of the same kind, with a stable deployment and interference-free process.

## 1. Introduction

As satellite-communication-related technologies continue to prosper, spaceborne antennas (usually deployable) are confronted with great challenges regarding the increasing demand for higher frequency in diverse fields, which involve but are not limited to telemetry, tracking and control (TT&C), remote sensing, radio astronomy, etc. During the past several decades, cable network antennas (CNAs) [1,2,3,4,5,6,7,8,9], represented by AstroMesh [10], have been dominating among several deployable antenna concepts due to their distinctive advantages of being lightweight and having a high stowing efficiency. According to the existing studies, the surface accuracy of these antennas usually stays at the millimeter level [1,11,12,13,14,15,16], corresponding to the L-S operating frequency band. If adopting various delicate structures (such as an umbrella-type structure [17,18,19,20]) or sophisticated adjustment approaches [11,12,13,16,21,22,23,24], small-aperture CNAs are capable of achieving an accuracy of 0.3–1 mm, with which its maximum operating frequency can reach the Ka band. However, the requirement for antenna operating frequency has been increasing invariably, which has far exceeded the Ka band, and even increased to the terahertz band. According to Ruze’s formulation [25], if the antenna operating frequency exceeds the Ka band with 5% gain loss, the antenna surface accuracy shall be no less than 0.125 mm and/or even reach the micron level if the operating frequency is in the terahertz band. As a result, the CNA becomes inappropriate for higher frequency beyond the Ka band. In view of the above challenges, the long-neglected rigid-reflector spaceborne antennas (RRSAs) perfectly fit this situation since the accuracy of the panels composing their reflectors can be machined to the micrometer level using modern processing techniques. This extraordinary accuracy guarantees the highest operating frequency attaining the terahertz band [26,27].

Several kinds of RRSAs had been proposed since the 20th century, which differ in the segmentation of their reflective surfaces. Specifically, their types can be categorized as the sunflower [28], the solid surface deployable antenna (SSDA) [29,30] and the petal-type deployable antenna (PTDA) [26,31,32,33,34,35,36,37,38], respectively. Among them, the former two types, ever since they were first proposed, drew little interest due to their various intrinsic structural deficiencies involving but not limited to complicated structures, lack of back frame, etc. In contrast to those two types, the third one has been most widely adopted owing to its maintainability, element universality and mutual dynamic balance in deployment [39]. Regarding its segmentation scheme, the reflector of the PTDA is segmented into multiple identical parts (usually known as petals) in the radial direction, with the rest in the center functioning as the central hub.

Despite several companies and universities proposing their exclusive PTDAs and manufacturing the corresponding experimental prototypes, respectively [28,31,32,40], none of them had been implemented in practical application scenarios so far. In 2011, Russia successfully launched the Spektr-R satellite (RadioAstron) [34,41,42] to conduct interferometer observation tasks. Combined with the global ground radio telescope network, the satellite can achieve extraordinarily high angular resolution to obtain images, coordinates, motions and evolution of the angular structure of different radio-emitting objects in the Universe. The antenna of the satellite is characterized with a 10 m diameter and 3.33 deploy/fold ratio, and was the largest space-based rigid-reflector antenna ever recorded. In addition, its reflector is composed of 27 carbon-fiber petals together with a central dish, and its 0.3 mm surface accuracy enables its operating frequency to reach up to the Ka band. According to the related literature, its deployment is conducted by rotating all the petals around their respective skew axis [39]. Unfortunately, no implementation details can be found involving the method for identifying the axis position, the deployment mechanism, the synchronous scheme, etc. Encouraged by the success of the Spektr-R project, Russia initiated its observatory-class mission, named Spektr-M (Millimetron) [26,27]. The Spektr-M is a 10 m space telescope designed to further investigate various objects in the Universe at millimeter and infrared wavelengths from 0.07 mm to 10 mm. Its antenna is of the same size as the Spektr-R, but its number of petals is reduced to 24, thereby enabling stronger deployment reliability. According to the existing literature [43], deploying the antenna of the Spektr-M is also accomplished by rotating all of its petals around their respective oblique axis in space. Similar to the Spektr-R, its specific implementation also remains unavailable nevertheless.

One of the most serious deficiencies of the above PTDA is its considerably low stowing efficiency. For example, the deploy/fold ratios of RadioAstron and MilliMetron are merely 3.33, but that of the CNA can reach up to 10 to 50 [1,2,3,4,5,6,7,8,9]. This inevitably results in smaller-sized antenna stowed in rocket fairing and eventually in poor electromagnetic (EM) performances in terms of gain, resolution and sensitivity. The most straightforward approach to improving the stowing efficiency of the PTDA is to increase the number of petals. According to the reliability theory [44], however, the deployment process can be viewed as a serial system, which suggests that more petals implies not only poorer deployment reliability but also a more serious position error and larger gap among deployed petals. Therefore, the above approach is inadvisable for realizing high stowing efficiency. The second approach improving the stowing efficiency is conducted through the conjunction of the pose adjustment and bisection method [45]. This approach improves the stowing efficiency to some extent without increasing the petals, and the efficiency can be further improved if adopting what is called the deviation-angle panel proposed in this study.

In addition to the abovementioned problems and deficiencies, deployment dynamics are also of critical importance for identifying appropriate structural parameters to achieve excellent dynamical performance, to which only a few studies have paid attention. Huang et al. [32,36] conducted the related dynamics research on small-sized RRSAs driven by torsional springs. Guang et al. [46] analyzed and experimented on the deployment process of a tiny 3D-printing antenna model based on rigid modified origami flashers. Other studies [28,29,38,39] place the emphasis on antenna concepts, mechanism configuration synthesis and kinematics. Therefore, there are still many directions worthy of in-depth research on the deployment dynamics of RRSAs.

In response to the aforementioned problems and challenges, we propose a novel type of RRSA based on PTDAs in this study, which is characterized with an ingenious segmentation scheme to realize higher stowing efficiency. Furthermore, we also investigate the matching antenna configuration, the deployment principle, the mechanism/structure and the deployment dynamics in order to ensure an interference-free and stable deployment process. The remainder of this paper is organized as follows. Section 2 reviews the existing concepts of RRSAs and analyzes their distinctive features. Section 3 presents an ingenious segmentation scheme of the proposed RRSA with a higher stowing efficiency than the traditional ones. Section 4 outlines the configuration design based on parameter identification for the optimal stowing efficiency, verifying the interference-free deployment preliminarily. Section 5 formulates the structure and deployment mechanism design of a Cassegrain RRSA for subsequent investigation of deployment dynamics. Section 6 highlights the study of the antenna deployment dynamics through trajectory planning. Section 7 summarizes conclusions of this paper.

## 2. Feature Analysis of Existing RRSA Concepts

### 2.1. Description of RRSA

**Figure 1:**
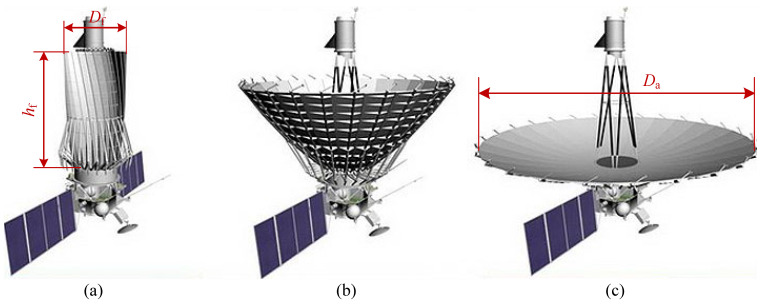
Deployment of Spektr-R. (**a**) Folded state. (**b**) Intermediate state. (**c**) Deployed state. The RSE is defined as $D_{a}/D_{f}$ and VSE is defined as $D_{a}/h_{f}$ .

For ease of subsequent reference and quotation, we present a simple description of the rigid-reflector spaceborne antenna in this subsection. Figure 1 shows the process of deploying the antenna of the Spektr-R satellite after unfolding its solar array. Similar to the Spektr-R, the reflective surface of a typical RRSA usually consists of circumferential panels and a central hub. In the folded state, all of the panels are around the hub and above it in a circular arrangement. Therefore, the configuration profile of the antenna can be approximated as a cylinder.

With respect to the cylinder profile, the ratio of aperture diameter to the maximum radial profile dimension of the antenna is referred to as the radial stowing efficiency (RSE). For instance, for the particular antenna of Spektr-R, its RSE is the deploy/fold ratio $D_{a}/D_{f}$ . In addition, the ratio from aperture diameter to the vertical profile dimension is defined as the vertical stowing efficiency (VSE). For the Spektr-R, the VSE is $D_{a}/h_{f}$ . If both RSE and VSE are large, the overall degree of system compactness is high. Unfortunately, these two values contradict each other, between which certain trade-offs must be made. This suggests that larger RSE implies lower VSE, and vice versa. In reality, the primary concern in practice is the RSE rather than the VSE, since manufacturing large-aperture fairing is far more difficult than manufacturing long fairing. For example, China’s Long March-5B rocket possesses a fairing with a 5.2 m diameter and 20.5 m length [47]. Therefore, one advisable strategy is ensuring the RSE first and then improving the VSE as much as possible. In this research, RSE is the sole pursued index for convenience of discussion. To sum up, the following investigation primarily focuses on the RSE of the RRSA, and whenever the stowing efficiency is mentioned in this paper, it defaults to RSE.

There are basically three concepts of the RRSA regarding the segmentation of the reflective surface of the antenna. The below subsection introduces these concepts and conducts a systematic analysis with respect to their characteristics, thereby forming a new RRSA concept. It has been acknowledged that the segmentation can be abstracted as follows. Firstly, the reflective surface is segmented into n(≥ 6) identical units (axisymmetric elements) in the circumferential direction, and each unit is sub-segmented into m(≥ 1) panels, with the rest performing as the central hub. Obviously, smaller *m* suggests higher axial symmetry.

### 2.2. Sunflower

**Figure 2:**
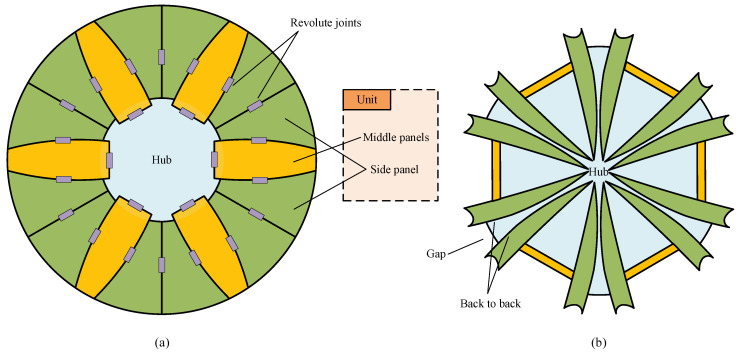
Sunflower antenna. (**a**) Deployed state. (**b**) Folded state. This antenna has 6 units, each of which has 3 panels.

The basic deployment concept was initially developed by the TRW company to satisfy the requirement for large-diameter (D/λ>1000) , high-accuracy reflectors to be used in the 10 GHz to 100 GHz range or beyond, within the constraints of the shuttle [28]. In the deployed state shown in Figure 2a, the sunflower’s reflective surface is split into six units in the circumferential direction, with a hub left in the center (blue). Each unit is sub-segmented into two side panels (green) and a middle panel (yellow), and all the panels and the hub are connected with revolute joints that are represented by the purple rectangles in the figure. When folded by the revolute joints and the synchronous equipment (not drawn), the overall profile can be readily stowed in a cylindrical rocket fairing, as shown in Figure 2a. In the folded configuration, all the side panels are erected, pointing into the hub center. Meanwhile, all the main panes are sandwiched by their respective side panels and are tangent to the circumferential direction in a way that looks like a regular hexagon. The number of units and the panel number of each unit can be increased to obtain a higher stowing efficiency. However, the deployment reliability may decrease accordingly due to the more complex structure.

It can be observed that the adjacent side panels are folded in a back-to-back manner; it is therefore unfeasible to introduce the supporting structures in the gaps to provide high surface accuracy, since the interference may be induced among supporting structures. Although the above concept is often reported in various literature, the deployment details of the sunflower antenna still remain unclear. In order to further study this concept, some efforts have been made by researchers in China. Guo et al. conducted relatively comprehensive research on its design and analysis [48,49]. Impressively, two linked Bennett mechanisms in symmetric form (known as the ‘Twin-Bennett mechanism’) were used to fulfill the deployment [50]. Zeng et al. improved this concept in its segmentation scheme to achieve higher stowing efficiency [30]. Also, they implemented the deployment by means of Twin-Bennett mechanisms. In addition, Wang et al. [51] reported the same deployment mechanism adopting the ‘Square-Twist pattern’ according to the principle of rigid origami [52]. To sum up, this concept does not draw much interest for various reasons and is not a compelling choice for high-frequency antennas.

### 2.3. Solid Surface Deployable Antenna (SSDA)

**Figure 3:**
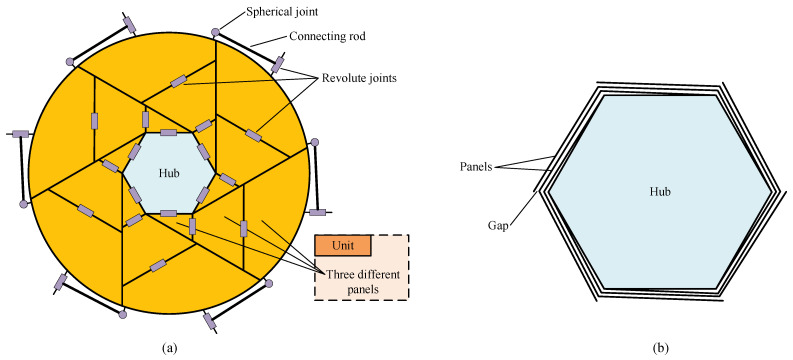
Solid surface deployable antenna. (**a**) Deployed state. (**b**) Folded state. This antenna has 6 units, each of which has 3 panels.

The concept of the solid surface deployable antenna (SSDA) was proposed by Guest at Cambridge University in 1996 [29], to which its detailed segmentation scheme is demonstrated in Figure 3. It can be observed from the figure that, for the SSDA, its central hub is a hexagonal panel, around which six identical wings are mounted, with each one being subdivided into three panels. When the SSDA is folded, all of its wings warp around the central hub, as shown in Figure 3b. The adjacent panels are linked by revolute joints, and all the wings are linked to the central hub by revolute joints, too. To serve the purpose of one degree-of-freedom (DOF) of the mechanism, additional connecting rods are installed between adjacent wings, as illustrated in Figure 3a. In its folded state, we can see that the gap between panels is considerably small, which precludes the possibility of introducing the supporting structures for a higher surface accuracy. To realize synchronous deployment, the number of motors shall be the same as that of the units, to which strictly synchronizing those motors is also required.

The SSDA concept is similar to the sunflower concept: its units and panels can also be increased to achieve higher stowing efficiency, under which circumstance the structure complexity, position error of each panel and number of driving motors will rise drastically. As a result, an antenna with low deployment reliability and surface accuracy is less meaningful, and this antenna concept has also been neglected for a long time, where only a few scholars have proceeded with the research. Luo et al. analyzed the correlation among wing number, panel number of each wing and structural parameters [53]. Eventually, they presented the formulations of the maximum edge length of the central hub and panel number. They concluded that the configuration that remains the same as that in Figure 3 is preferable given various factors.

### 2.4. Regular-Panel Antenna

**Figure 4:**
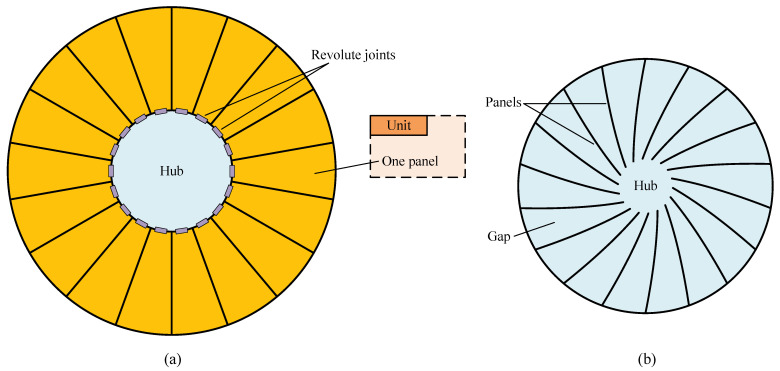
Petal-type deployable antenna. (**a**) Deployed state. (**b**) Folded state. (1) This antenna has 18 units, each of which has only one panel; (2) The position of the revolute joint must be finely arranged for precise antenna deployment.

With respect to the regular-panel antenna concept, i.e., the PTDA mentioned in Section 1, its reflective surface is segmented into a series of identical panels in the radial direction, with a circle-shaped hub left in the center, as shown in Figure 4a. Its unfolding process is like a blooming flower; hence, it is referred to as the petal-type deployable antenna [32,36,39], and the panel together with its supporting structure (framework) is usually called a petal. All the panels are fixed on their respective framework and can be easily linked to the hub with tilted revolute joints (purple rectangles) through these frameworks, so the deployed or folded PTDA can be implemented by rotating the panels around their respective joint axes. This scheme has been widely adopted by many designs [31,32,33,35,36], owing to its structure simplicity. Some designs introduced a base to connect the panel [38]. No matter what schemes are adopted, the structure of the PTDA exhibits high axisymmetry at any phase of deploying or folding, possessing excellent commonality of elements in deployment balance [39] compared with the aforementioned antenna concepts. In the folded state, all the panels form a pattern similar to the vertical view of a fan impeller, as shown in Figure 4b.

The gap between adjacent panels is large enough, and the back of one panel is exactly opposite to the face of another, thereby ensuring a well-suited gap for accommodating satisfactory supporting structures to realize high surface accuracy. For instance, the surface accuracy of the FIRST antenna [31] and Millimetron antenna are 8μm and 6μm , respectively. In terms of manufacturing, only two sets of molds are required for fabricating all the panels, where, by adopting them, the extra economic merit of this antenna type outperforms those of the other two.

The above advantages make this antenna concept become the most prevailing one among those three antenna concepts, for which the characteristics of the above concepts are listed in the first three rows of Table 1 for a comprehensive comparison.

Although three commonly adopted RRSA concepts exist, their RSEs are still low. According to Table 1, the regular panel is the preferred concept, based on which we propose a new antenna concept while utilizing the advantages of the regular panel in subsequent sections.

## 3. Deviation-Angle-Panel-Based Antenna Concept

This section presents a new RRSA concept with a higher stowing efficiency that utilizes most of the advantages of the PTDA. It is inspired by the process of improving the stowing efficiency of the PTDA by adjusting the panel pose (position and orientation).

### 3.1. Deviation-Angle Panel

**Figure 5:**
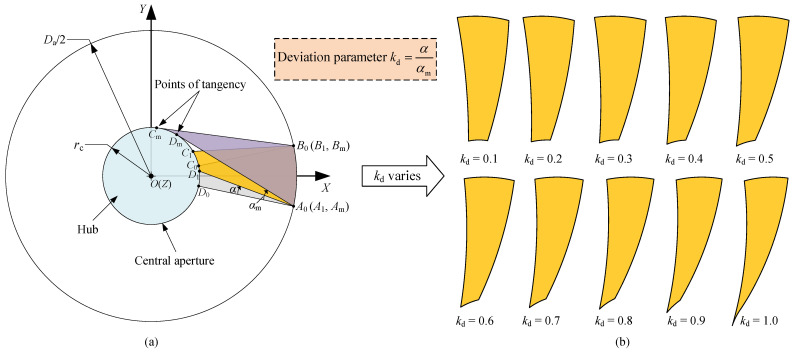
Segmentation scheme of deviation-angle panel. (**a**) Top view. (**b**) Deviation-angle panels. The regular panel $A_{0}B_{0}C_{0}D_{0}$ , colored in gray, is obtained by dividing the reflective surface into *n* identical circumferential parts in radial direction, leaving a circular hub at the center.

Figure 5a shows the top view of a deviation-angle panel. To reduce the magnitude of $∠B_{0}A_{0}D_{0}$ , deviate the radial line segment $A_{0}D_{0}$ from the original position by angle α (deviation angle) to obtain a new panel $A_{1}B_{1}C_{1}D_{1}$ , which is colored in yellow. In this way, if the value of $∠B_{0}A_{0}D_{0}$ on plane OXY decreases, so does the actual $∠B_{0}A_{0}D_{0}$ in space. If the deviation angle α continues to increase until line segment $A_{1}D_{1}$ is tangent to the central aperture, then line segment $A_{1}D_{1}$ will reach its limit position $A_{m}D_{m}$ , and α obtains its maximum $\alpha_{m}$ , thereby obtaining the limit panel $A_{m}B_{m}C_{m}D_{m}$ , colored in purple. Let the deviation angle $\alpha =k_{d}\alpha_{m}$ , where $k_{d}\in \left[0,1\right]$ . If $k_{d}=0$ , the deviation angle is 0, if $k_{d}=1$ , the deviation angle is $\alpha_{m}$ and if $0<k_{d}<1$ , the deviation angle is between 0 and $\alpha_{m}$ . Therefore, parameter $k_{d}$ is referred to as the deviation parameter. It is evident that the case $k_{d}=0$ corresponds to the regular panel; thus, our proposed panel is a generality of the regular panel described in Section 2.4, which is referred to as the deviation-angle panel. This type of segmentation scheme retains many advantages possessed by the regular one. For instance, all the deviation-angle panels are the same, and both the deployed and the folded configuration are axisymmetric about the *Z*-axis, exhibiting higher axisymmetry than other antenna concepts such as the sunflower concept, since the unit of the sunflower has three panels whereas the new segmentation scheme has only one panel. Additionally, because all panels are identical, the newly obtained panel has a lower manufacturing cost compared with other segmentation schemes such as the sunflower and SSDA; hence, only one set of mold is required for fabricating it. All of the specifications are listed in the last row of Table 1.

### 3.2. Improving RSE by Pose Adjustment

Referring to the second scheme improving the RSE in Ref. [45], the antenna RSE can be improved by simply rotating the regular panel. If combined with the deviation-angle panel, performing the same scheme can further improve the RSE, which is elaborated as follows.

**Figure 6:**
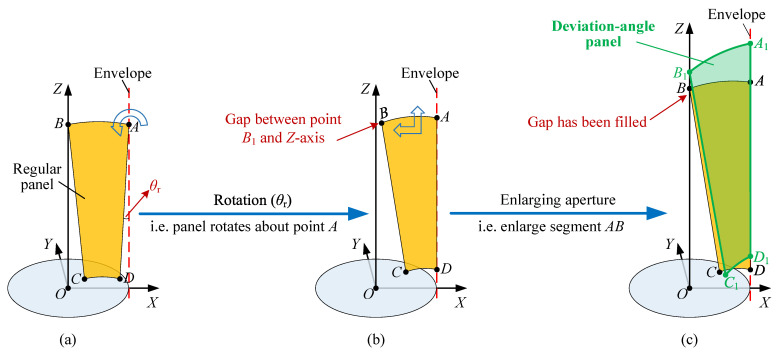
Process of pose adjustment. (**a**) Initial pose. (**b**) Intermediate pose. (**c**) Final pose.

As shown in Figure 6a, the regular panel is in its initial pose, in which its three corner points A,B,C are located on plane OXZ , and line segment AB is parallel to plane OXY , with points *A* and *B* precisely on the envelope and *Z*-axis, respectively. Exert a rotation by $\theta_{r}$ on the panel about *A* until *D* is on the envelope to obtain the intermediate pose of the regular panel, as shown in Figure 6b. This pose creates a gap between point *B* and the *Z*-axis. Then, enlarging the aperture until the gap is filled will increase the RSE, as illustrated in Figure 6c. It can be observed from Figure 6a,b that a smaller ∠BAD induces a larger angle $\theta_{r}$ and gap and, eventually, a higher RSE. Since the angle ( ∠BAD ) is of critical importance in improving the RSE, we name it as the key angle (KA). Note that the magnitude of the KA cannot be changed by adjusting the pose because it stays fixed if the number *n* of regular panels is given. According to Figure 5, the KA decreases with an increase in $k_{d}$ .

Hence, the KA can be reduced if the regular panel is in place with the deviation-angle panel. As shown in Figure 6c, the green panel $A_{1}B_{1}C_{1}D_{1}$ is the deviation-angle panel, and it is obvious that its KA ( $∠B_{1}A_{1}D_{1}$ ) is smaller than that of the regular panel. Thus, the length of line segment $A_{1}B_{1}$ is greater than that of AB , which means that the RSE of the antenna with the deviation-angle panel is greater than that with the regular panel. For ease of expression, hereinafter, the pose of the deviation-angle panel in Figure 6c is named as the compact pose (CP).

### 3.3. Rotation Angle

From the immediate geometric relation in Figure 5a, we can obtain

(1) $$\alpha_{m}=\arcsin\frac{r_{c}}{\frac{D_{a}}{2}}=\arcsin\frac{2r_{c}}{D_{a}}.$$

It can be observed from Figure 5b that the deviation parameter $k_{d}$ increases and the panel becomes longer in height and narrower at the bottom, causing a decrease in VSE. However, as mentioned earlier, the height of the rocket fairing is much greater than its diameter, so this phenomenon has little influence on the storage of the antenna. In Figure 5a, assume that the polar angle of point $D_{1}$ is $\theta_{D_{1}}$ ; thus, its position vector on plane OXY is ${\left(r_{c}\cos\theta_{D_{1}},r_{c}\sin\theta_{D_{1}}\right)}^{T}$ , and the position vector of point $C_{1}$ on plane OXY is ${\left(r_{c}\cos\left(\theta_{D_{1}}+\frac{2\pi }{n}\right),r_{c}\sin\left(\theta_{D_{1}}+\frac{2\pi }{n}\right)\right)}^{T}$ , where $\theta_{D_{1}}$ is an unknown to be determined ( $\theta_{D_{1}}$ will be used in the next section). Referring to the law of sines [54], we have

(2) $${\bar{l}}_{OD_{1}}\sin\left(\theta_{D_{1}}-\theta_{A_{1}}\right)={\bar{l}}_{A_{1}D_{1}}\sin\left(k\alpha_{m}\right),$$

where ${\bar{l}}_{OD_{1}}$ and ${\bar{l}}_{AD_{1}}$ are the lengths of projections of line segments $OD_{1}$ and $A_{1}D_{1}$ on plane OXY , respectively, and $\theta_{A_{1}}$ and $\theta_{D_{1}}$ are corresponding polar angles of points $A_{1}$ and $D_{1}$ , as well as $\theta_{A_{1}}=-\frac{\pi }{n}$ . Meanwhile, the explicit expressions of ${\bar{l}}_{OD_{1}}$ and ${\bar{l}}_{A_{1}D_{1}}$ are

(3) $$\left\{\begin{array}{c} {\bar{l}}_{OD_{1}}=r_{c},\\ {\bar{l}}_{A_{1}D_{1}}=\sqrt{{\left(\frac{D_{a}}{2}\cos\frac{-\pi }{n}-r_{c}\cos\theta_{D_{1}}\right)}^{2}+{\left(\frac{D_{a}}{2}\sin\frac{-\pi }{n}-r_{c}\sin\theta_{D_{1}}\right)}^{2}}, \end{array}\right.$$

respectively. It can be found from Equations (2) and (3) that the equation about $\theta_{D_{1}}$ is a complex nonlinear equation, so Newton’s method [55] can be applied here to solve it. Solving Equation (2) obtains $\theta_{D_{1}}$ and then the position vector of point $D_{1}$ . According to the manipulation demonstrated in Figure 6, the rotation angle $\theta_{r}$ can be expressed as

(4) $$\theta_{r}=\frac{\pi }{2}-\arccos\frac{r_{A_{1}D_{1}}\cdot r_{A_{1}B_{1}}}{|r_{A_{1}D_{1}}\left|\right|r_{A_{1}B_{1}}|}.$$

## 4. Parameter-Identification-Based Configuration Design

Since the configuration of all panels is the skeleton of the overall antenna structure and the deployed configuration is known, the folded configuration should be studied first when proceeding with our subsequent investigation on the structure and deployment mechanism. This section presents a detailed discussion concerning the optimal stowing configuration of the antenna. (For ease of expression, hereinafter, the word ’configuration’ refers to the default folded configuration of the antenna.)

### 4.1. Deployment Implementation and Theoretical RSE

**Figure 7:**
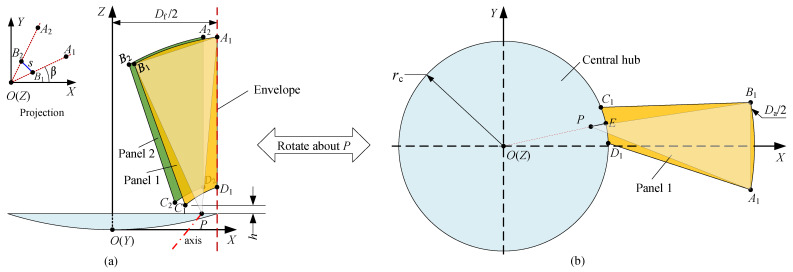
Deployment implementation based on Euler’s rotation theorem. (**a**) Front view of folded state. (**b**) Top view of deployed state. (1) The folded radius $\frac{D_{f}}{2}$ is taken the same as radius $r_{c}$ of the central aperture for high VSE; (2) the panel is deployed or folded by the rotation about the fixed point *P*, whose position is elaborately formulated to fulfill the constraints.

We have mentioned in Section 2.4 that most PTDAs are deployed with the rotation about fixed oblique axes in space, and this also applies to the antenna proposed in this study. Here, we utilize Euler’s rotation theorem [56,57] to determine the position of the rotation axis. Firstly, a fixed point *P* is determined, about which the panel will rotate. Then, according to Euler’s rotation theorem, there exists such an axis passing through the point that the rotation can be realized by a single rotation around the axis, as illustrated in Figure 7. However, the situation differs due to the adopted panel pose, which is the same as that in Figure 6c for high stowing efficiency, as shown in Figure 7a. Apart from this, radius $r_{c}$ is taken as the same magnitude as folded radius $\frac{D_{f}}{2}$ to improve the VSE, as shown in Figure 7a. This manipulation also contributes to the improvement in RSE because a larger $r_{c}$ implies not only a larger $\alpha_{m}$ in accordance with Equation (1) but eventually a higher RSE according to the process of increasing RSE in Figure 6. These differences from Ref. [45] bring additional and more complex constraints, which are elaborated as follows.

As shown in Figure 7b, taking panel 1 as an example, the deployed panel is transformed into the folded one in Figure 7a by rotating about the fixed point *P*, and the pose of the folded panel is the same as that of the green panel in Figure 6c. Specifically, the following four geometric constraints (GCs) can be summarized.

1. Point $A_{1}$ is on the envelope (GC 1), and it is conceivable that this constraint is easy to fulfill since line segment $PA_{1}$ always intersects with the envelope at point $A_{1}$ after an appropriate rotation about *P*.
2. Under the first constraint, locating point $D_{1}$ precisely on the envelope (GC 2) is difficult to realize because the two constraints are coupled.
3. Point $C_{1}$ has a prespecified upward displacement *h* (GC 3) to avoid interference between the panel and central surface. This constraint should be fulfilled under the first two constraints.
4. According to Figure 6c, the focal axis, i.e., coordinate axis OZ , and plane $A_{1}B_{1}D_{1}$ are coplanar (GC 4). This constraint should be satisfied under the former three GCs. Thus, we lower the requirement first and simply guarantee that axis OZ is coplanar with points $A_{1}$ and $B_{1}$ (weak GC 4). When points $A_{1}$ and $B_{1}$ are projected onto plane OXY , their polar angles are the same, denoted as β , which is a parameter to be determined, as shown in the top-left subfigure of Figure 7a. In addition, to avoid interference caused by the panel thickness, it is assumed that the distance between $B_{1}$ and $B_{2}$ is a prespecified parameter *s*, named an interference parameter.

In summary, the first constraint can be directly satisfied, but the rest of the constraints cause a problem in identifying a proper fixed point *P* to fulfill themselves. In response to this problem, an ingenious scheme will be formulated and expanded on in Section 4.2. We now return to Figure 7 and assume that all of the four GCs are satisfied. Based on its immediate geometric relation, we arrive at the following expression:

(5) $$l_{A_{1}B_{1}}\cos\theta_{r}+l_{OB_{1}}=\frac{D_{f}}{2},$$

where $l_{A_{1}B_{1}}=D_{a}\sin\frac{\pi }{n}$ and $l_{OB_{1}}=\frac{s}{2\sin\frac{\pi }{n}}$ . Solving Equations (2) to (5) yields the theoretically maximum $D_{a}$ , denoted as $D_{a}^{t}$ , and hence the theoretical RSE is expressed as follows:

(6) $$\zeta_{t}=\frac{D_{a}^{t}}{D_{f}}.$$

### 4.2. Parametric Error Compensation Model

**Figure 8:**
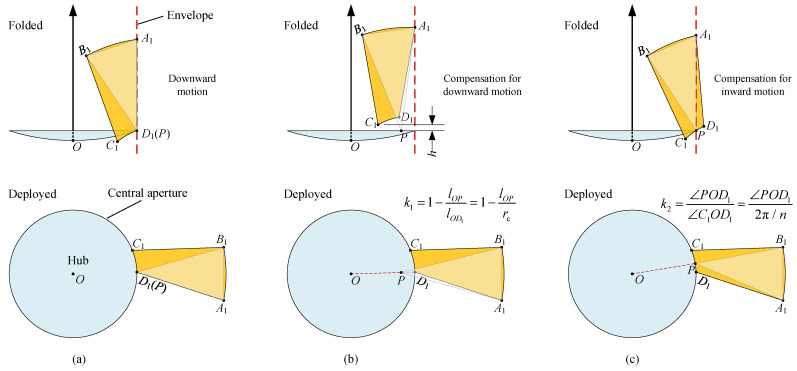
Functions of $k_{1}$ and $k_{2}$ . (**a**) Without parameters. (**b**) Function of parameter $k_{1}$ . (**c**) Function of parameter $k_{2}$ . (1) Parameter $k_{1}$ can compensate for the downward motion of $C_{1}$ in subfigure (**a**); (2) parameter $k_{2}$ can compensate for the inward motion of $D_{1}$ in subfigure (**b**); (3) if the values of $k_{1}$ and $k_{2}$ are appropriately prescribed, both constraints can be satisfied.

From the discussion above, we know that it is unfeasible to identify the precise position of the fixed point *P* directly. As a result, we therefore begin with a special position of *P* and gradually approach the expected solution, adopting the idea of parameter identification in robotics [58,59]. Parameter identification is the process of minimizing the residuals between observed data and corresponding parametric error compensation model (PECM). In order to exploit the idea of parameter identification, the geometric model in Figure 7, which completely fulfills all of the GCs, is regarded as the observed data. To fit the observed data, a PECM is established first. We begin with a simple example in which the fixed point *P* is located at a special position, as shown in Figure 8a. In the deployed state, *P* coincides with $D_{1}$ , points $A_{1}$ and $D_{1}$ are precisely located on the envelope after folding and the focal axis is coplanar with points $A_{1}$ and $B_{1}$ , suggesting that GCs 1 and 2 as well as the weak GC 4 are satisfied. However, point $C_{1}$ fails to obtain an upward displacement *h*; instead, it has a downward motion and interferes with the central hub. To compensate for this error, the position of *P* is relaxed by moving a certain distance in the radial direction on the plane of the central aperture. Meanwhile, a parameter $k_{1}$ is introduced here to quantify the compensating effect, as shown in Figure 8b. In this way, it is conceivable that point $C_{1}$ will have an upward motion, and that a larger $k_{1}$ implies that the upward motion is more noticeable.

Unfortunately, the above operation will inevitably introduce another error, where point $D_{1}$ is not exactly located on the envelope; instead, it will be completely inside the envelope. This panel pose differentiates from that described in Figure 6c, which eventually brings about a descent in RSE. To additionally compensate for this error, another compensating strategy is introduced here. As shown in Figure 8c, the compensation parameter $k_{2}$ is defined as $k_{2}=∠POD_{1}/∠C_{1}OD_{1}$ , and, by definition, the location of the fixed point *P* is equivalent to traveling a certain path relative to point $D_{1}$ anticlockwise along arc $C_{1}D_{1}$ , as shown by the bottom subfigure in Figure 8c. After folding, point $D_{1}$ will possess an outward motion and even pass through the envelope to be completely outside it, as shown in the top subfigure of Figure 8c. Except for $k_{1}$ and $k_{2}$ , parameter β in Figure 7a also affects the pose of the panel, and GC 4 can be satisfied if assigning it an appropriate value.

Given the compensating effect of parameters $k_{1}$ , $k_{2}$ and β , they are collectively referred to as the compensation parameters. According to the discussion above, these compensation parameters are capable of characterizing the deviation of the nominal values and observed values, thereby verifying the completeness of the PECM. Furthermore, small changes in the panel pose must correspond to small changes in the compensation parameters, by which the continuity of the model can be validated. Note that, $k_{1}$ , $k_{2}$ and β are independent of each other, and thus the PECM has a minimal number of parameters; namely, it possesses the property of minimality. In summary, the PECM exhibits three distinguishing properties of completeness, continuity and minimality, thereby satisfying the basic requirements for parameter identification [60,61] (see Appendix A for a detailed explanation on the PECM properties of completeness, continuity and minimality). Therefore, all of the above four GCs will be satisfied if proper values are assigned to those compensation parameters.

### 4.3. Parameter Identification of Configuration

It can be observed from Figure 7 and Figure 8 that the position vector of the fixed point *P* can be expressed as the following form:

(7) $$r_{P}={\left(\left(1-k_{1}\right)r_{c}\cos\left(k_{2}\frac{2\pi }{n}+\theta_{D_{1}}\right),\left(1-k_{1}\right)r_{c}\sin\left(k_{2}\frac{2\pi }{n}+\theta_{D_{1}}\right),\frac{r_{c}^{2}}{4f},1\right)}^{T},$$

where *f* is the focal length and equal to $\eta D_{a}$ ( η is the focus/diameter ratio). It is commonly known that three non-collinear points in space determine a rigid body and its pose naturally. According to the discussion above, the deployed pose can be represented by point set $\left\{A_{1},B_{1},P\right\}$ , and their (including point $C_{1}$ ) corresponding position vectors (or coordinates) are   

(8) $$\left\{\begin{array}{c} r_{A_{1}}={\left(\frac{D_{a}}{2}\cos\frac{-\pi }{n},\frac{D_{a}}{2}\sin\frac{-\pi }{n},\frac{r_{c}^{2}}{4f},1\right)}^{T},\\ r_{B_{1}}={\left(\frac{D_{a}}{2}\cos\frac{\pi }{n},\frac{D_{a}}{2}\sin\frac{\pi }{n},\frac{r_{c}^{2}}{4f},1\right)}^{T},\\ r_{P}={\left(\left(1-k_{1}\right)r_{c}\cos\left(k_{2}\frac{2\pi }{n}+\theta_{D_{1}}\right),\left(1-k_{1}\right)r_{c}\sin\left(k_{2}\frac{2\pi }{n}+\theta_{D_{1}}\right),\frac{r_{c}^{2}}{4f},1\right)}^{T},\\ r_{C_{1}}={\left(r_{c}\cos\left(\frac{2\pi }{n}+\theta_{D_{1}}\right),r_{c}\sin\left(\frac{2\pi }{n}+\theta_{D_{1}}\right),\frac{r_{c}^{2}}{4f},1\right)}^{T}, \end{array}\right.$$

respectively. On the other hand, the folded pose in Figure 7a can be represented by the corresponding point set, denoted as ${\left\{A_{1},B_{1},P\right\}}^{\prime }$ . In light of Figure 7 and Figure 8, the coordinates of ${\left\{A_{1},B_{1},P\right\}}^{\prime }$ can be expressed as

(9) $$\left\{\begin{array}{c} r_{A_{1}}^{\prime }={\left(\frac{D_{f}}{2}\cos\beta ,\frac{D_{f}}{2}\sin\beta ,z_{A_{1}},1\right)}^{T},\\ r_{B_{1}}^{\prime }={\left(r_{B_{1}}\cos\beta ,r_{B_{1}}\sin\beta ,z_{B_{1}},1\right)}^{T},\\ r_{P}^{\prime }={\left(\left(1-k_{1}\right)r_{c}\cos\left(k_{2}\frac{2\pi }{n}+\theta_{D_{1}}\right),\left(1-k_{1}\right)r_{c}\sin\left(k_{2}\frac{2\pi }{n}+\theta_{D_{1}}\right),\frac{r_{c}^{2}}{4f},1\right)}^{T}, \end{array}\right.$$

where $z_{A_{1}},r_{B_{1}}$ and $z_{B_{1}}$ remain unknown and shall be identified. Given that the panel is a rigid body, we have

(10) $$\left\{\begin{array}{c} {∥r_{A_{1}}-r_{B_{1}}∥}_{2}={∥r_{A_{1}}^{\prime }-r_{B_{1}}^{\prime }∥}_{2},\\ {∥r_{B_{1}}-r_{P}∥}_{2}={∥r_{B_{1}}^{\prime }-r_{P}^{\prime }∥}_{2},\\ {∥r_{P}-r_{A_{1}}∥}_{2}={∥r_{P}^{\prime }-r_{A_{1}}^{\prime }∥}_{2}. \end{array}\right.$$

Consequently, if values of $k_{1}$ , $k_{2}$ and β are given, all equations in Equation (10) can be solved simultaneously for unknowns $\left\{z_{A_{1}},r_{B_{1}},z_{B_{1}}\right\}$ . In this way, the deployed and folded poses of the panel can be totally determined, which can be represented by point sets $\left\{A_{1},B_{1},P\right\}$ and ${\left\{A_{1},B_{1},P\right\}}^{\prime }$ , respectively. This also suggests that a scenario is formed, which is suitable for using rigid-body registration algorithms [62,63] to obtain the deploying-to-folding transformation matrix denoted as T . Evidently, if β is given, the transformation matrix T is a function of $k_{1}$ and $k_{2}$ , and can be denoted as $g(k_{1},k_{2})$ . Consequently, our goal is to find such a transformation matrix $g(k_{1},k_{2})$ that, with its treatment, we arrive at

(11) $$\left\{\begin{array}{c} {\left(e_{1}g\left(k_{1},k_{2}\right)r_{D_{1}}\right)}^{2}+{\left(e_{2}g\left(k_{1},k_{2}\right)r_{D_{1}}\right)}^{2}-{\left(\frac{D_{f}}{2}\right)}^{2}=0,\\ e_{3}g\left(k_{1},k_{2}\right)r_{C_{1}}-\frac{r_{c}^{2}}{4f}-h=0, \end{array}\right.$$

where $e_{i}$ , i=1,2,3 , represents a vector whose *i*-th component is 1 and whose remaining components are all 0. It is fortunate that Equation (11) happens to have solutions. However, the process of error compensation is conducted according to our expectation, so there may not exist any solutions to Equation (11). Considering this situation, it is preferable to convert Equation (11) into an optimization model. Foremost, the error function can be expressed as follows:

(12) $$E\left(k_{1},k_{2}\right)={\left[{\left(e_{1}g\left(k_{1},k_{2}\right)r_{D_{1}}\right)}^{2}+{\left(e_{2}g\left(k_{1},k_{2}\right)r_{D_{1}}\right)}^{2}-{\left(\frac{D_{f}}{2}\right)}^{2}\right]}^{2}+{\left[e_{3}g\left(k_{1},k_{2}\right)r_{C_{1}}-\frac{r_{c}^{2}}{4f}-h\right]}^{2},$$

where $E(k_{1},k_{2})$ represents the residual produced by the left-hand side of Equation (11), which is a function of $k_{1}$ and $k_{2}$ . Secondly, as the inner optimization model, it can be formulated as follows:

(13) $$\left\{\begin{array}{c} \mathrm{find}\;(k_{1},k_{2}),\\ \min\;E(k_{1},k_{2}),\\ s.t.\;0<k_{1}<1,0<k_{2}<1, \end{array}\right.$$

If the minimum of the optimization problem in Equation (13) is 0 or a value considerably approximating 0, we obtain the precise solution of Equation (11); otherwise, we obtain a least-square solution.

In pursuit of the same pose as that described in Figure 6c, compensation parameter β is adjusted to maximize the distance from point $B_{1}$ to the *Z*-axis, thereby satisfying the GC 4; this fact will be verified in Section 4.4. This distance maximization can also be regarded as an optimization problem, so the objective function shall be established first. For a given β , its corresponding transformation matrix is obtained and denoted as T(β) , so the distance denoted as $g_{1}\left(\beta \right)$ can be formulated as:

(14) $$g_{1}\left(\beta \right)={∥\mathrm{Diag}\left(1,1,0,0\right)T\left(\beta \right)r_{B_{1}}∥}_{2},$$

where Diag(·) represents the diagonal matrix. Therefore, the outer optimization model can be described as

(15) $$\left\{\begin{array}{c} \mathrm{find}\;\beta ,\\ \max\;g_{1}\left(\beta \right),\\ s.t.\;\frac{-2\pi }{n}<\beta <\frac{2\pi }{n}, \end{array}\right.$$

where the maximum of the model is a function of $D_{a}$ , denoted as $g_{2}\left(D_{a}\right)$ . Eventually, finding the maximum aperture diameter $D_{a}$ is equivalent to solving the following equation:

(16) $$g_{2}\left(D_{a}\right)=\frac{s}{\sin\frac{\pi }{n}}.$$

After solving Equation (16), the maximum aperture diameter is denoted as $D_{a}^{*}$ , the resulting matrix is denoted as $T^{*}$ , the residual error is denoted as $E^{*}$ and the corresponding compensation parameters are denoted as $k_{1}^{*}$ , $k_{2}^{*}$ and $\beta^{*}$ , respectively. Furthermore, the resulting RSE, known as the simulated RSE and denoted as $\zeta_{s}$ , is equivalent to $D_{a}^{*}/D_{f}$ .

To sum up, the process of parameter identification is to solve a two-level nested optimization model (Equations (13) and (15)) in combination with an equation (Equation (16)), and its pseudocode is presented as follows. Thereinto, the inputs are the structural parameter values of the antenna and the outputs are the maximum aperture diameter, transform matrix and identified parameters. In addition, lines 1–6 are the definition of the residual function in Equation (12), lines 7–9 are the definition of the distance function in Equation (14), lines 11–14 are the definition of the maximum distance function in Equation (15) and line 15 finds the maximum aperture diameter.

Referring to Euler’s rotation theorem [56,57], the general displacement about a fixed point is equivalent to a single rotation about some axis passing through that point. In addition, the rotation angle and axial vector are

(17) $$\left\{\begin{array}{c} \theta_{d}=\arccos\frac{\mathrm{Tr}(R)-1}{2},\\ v=\mathrm{eig}(R,1), \end{array}\right.$$

respectively, where R denotes the corresponding rotation matrix contained in $T^{*}$ , Tr(·) denotes the operator used to obtain the trace of a matrix and eig(·,1) denotes the operator used to obtain the unit eigenvector corresponding to eigenvalue 1.

### 4.4. Effectiveness in Algorithm

**Figure 9:**
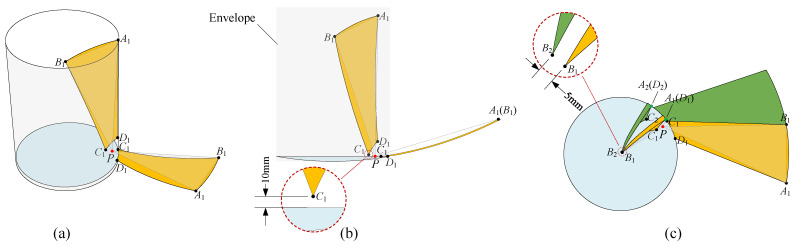
Verification of configuration. (**a**) Bimetric view. (**b**) Front view. (**c**) Top view.

In order to verify the effectiveness of the CPI algorithm in Algorithm 1, a concrete antenna configuration is investigated in this section. Thereinto, the initial aperture diameter $D_{a}$ is 2000 mm, the focus/diameter ratio η is 0.5, the number of panels is 18, the interference distance *s* is 5 mm, the vertical displacement *h* is 10 mm, the fairing diameter $D_{f}$ is 740 mm and the deviation parameter $k_{d}$ is 0.6. All values of the structural parameters are listed in the left-side column of Table 2. After executing the CPI algorithm, the relevant results are reported in the right-side column of Table 2, and the corresponding antenna configuration is shown in Figure 9. (The antenna configuration is represented in the same manner as that in Figure 7 for a visual and clear demonstration, which is generated in a precise way—programming in *Wolfram Mathematica*).
**Algorithm 1** Configuration parameter identification (CPI)
**Input:** Focus/diameter ratio η , number of panels *n*, fairing envelope diameter $D_{f}$ , deviation parameter $k_{d}$ , vertical displacement *h*, interference parameter *s*;
**Output:** Maximum aperture diameter $D_{a}^{*}$ , transformation matrix $T^{*}$ , residual $E^{*}$ , compensation parameters $k_{1}^{*},k_{2}^{*}$ and $\beta^{*}$ ;
1:**function** *E*( $k_{1},k_{2}$ )          ▹ Definition of residual function *E*2:    Give point sets $\left\{A_{1},B_{1},P\right\}$ and ${\left\{A_{1},B_{1},P\right\}}^{\prime }$ by solving Equation (10) using Newton’s method;3:    Give transformation matrix T from $\left\{A_{1},B_{1},P\right\}$ to ${\left\{A_{1},B_{1},P\right\}}^{\prime }$ by rigid-body registration algorithms;4:    Calculate the right-hand expression in Equation (12);5:    **return** *E*;6:**end function** 7:**function** $g_{1}$ ( β )           ▹ Definition of distance function $g_{1}$
8:    Calculate the right-hand expression in Equation (14); ▹ This step will repeatedly call function *E*9:    **return** $g_{1}$ ;10:**end function** 11:**function** $g_{2}$ ( $D_{a}$ )    ▹ Definition of maximum distance function $g_{2}$
12:    Solve the optimization model in Equation (15);  ▹ This step will repeatedly call functions *E* and $g_{1}$
13:    **return** $g_{2}$ ;14:**end function** 15:Solve Equation (16);   ▹ This step will repeatedly call function $E,g_{1}$ and $g_{2}$
16:**return** $D_{a}^{*}$ , $T^{*}$ , $E^{*}$ , $k_{1}^{*},k_{2}^{*}$ and $\beta^{*}$ ;  ▹ The expected axis can be obtained by $T^{*}$ , as elaborated below.


If the CPI algorithm is effective for the configuration, all of the four GCs in Section 4.1 must be satisfied first when the antenna is in its folded state. The following verification is performed by observation and measurement in *Mathematica*. Foremost, all of the GCs are confirmed.

The bimetric, front and top views demonstrating the above antenna folded configuration are presented in Figure 9, from which it can be observed in Figure 9a that points $A_{1}$ and $D_{1}$ are precisely located on the envelope, thereby satisfying GCs 1 and 2. In Figure 9b, it can be seen that point $C_{1}$ rises in distance after folding, and the displacement is 10 mm after measurement, by which GC 3 is therefore satisfied. From the top view in Figure 9c, line segment $A_{1}B_{1}$ is in the radial direction, and points $A_{1}$ and $D_{1}$ colored in green overlap entirely. Hence, the *Z*-axis is coplanar with points $A_{1},B_{1}$ and $D_{1}$ , which suggests that GC 4 is satisfied.

**Figure 10:**
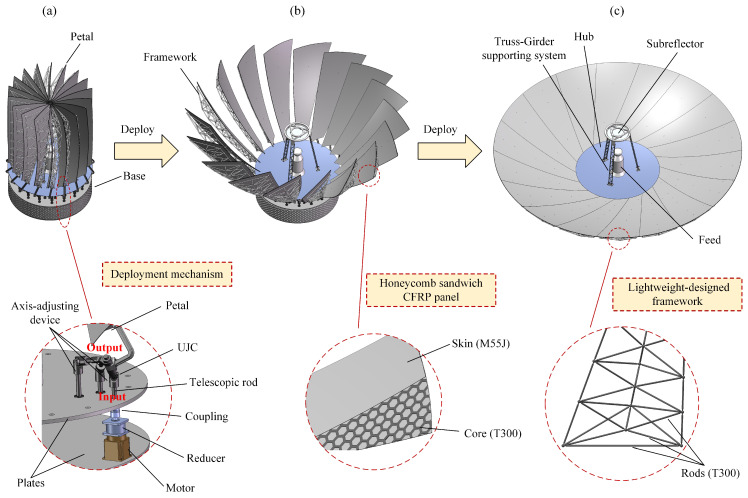
Structure design of Cassegrain antenna. (**a**) Folded state. (**b**) Intermediate state. (**c**) Deployed state. (1) The antenna structure primarily consists of honeycomb panels, trusses, hub, subreflector, deployment mechanisms, axis-adjusting device and base. (2) The structure composed of a panel and framework in subfigure (**a**) is called ’petal’.

In addition, it is found in *Mathematica* that the distance between points $B_{1}$ and $B_{2}$ is exactly 5 mm, and all the panels form the shape of a perfect paraboloid after deploying by an angle of $\theta_{d}=149.952^{{}^{\circ}}$ , as shown in Figure 10c. No interference was found during the deployment when simulating the deployment process in *Mathematica*. We also discovered in Table 2 that the theoretical and simulated RSE are identical, and that the residual $E^{*}$ approximates to 0. All the facts above verify the effectiveness in antenna configuration, proving the effectiveness of the parameter identification algorithm for this specific antenna.

Extensive simulations further suggest that the results are similar to those in Table 2 and Figure 9 if the structural parameters of the antenna are assigned with other values, thereby verifying the effectiveness of the configuration parameter identification algorithm for the antenna configuration presented in Algorithm 1.

### 4.5. Effectiveness in Stowing Efficiency

As claimed in Section 3, the newly proposed antenna featured with the deviation-angle panel and the configuration whose panel has the CP demonstrated in Figure 6c shall possess a higher RSE, which is one of its major advantages. Since the antenna’s folded configuration determines the RSE, it can be compared to the RSE of the proposed antenna. Therefore, we compared the RSE of the proposed antenna configuration with those of the existing RRSAs to verify the claim. The structural parameters of the existing RRSAs are listed in Table 3, and the comparison results are presented in Table 4. In order to further explore the RSE performance of the proposed antenna, the RSE obtained by utilizing the third RSE-improving scheme in Ref. [45] is reported in Table 5. In this scheme, the panel pose is obtained by conducting an optimization algorithm; hence, its RSE is higher than that in Table 4 theoretically.

According to Table 4, it is found that the RSE has been significantly improved. Thereinto, for the best one, the Dornier FIRST or Dornier MEA, its minimum improvement rate is 39.15% and the maximum reaches 46.70%. Even for the poorest one, the improved sunflower, its minimum improvement rate is 8.93% and its maximum attains 20.37%. For Millimetron, its minimum improvement rate is 16.17% and its maximum attains 22.47%. In addition, for each antenna in Table 4, the RSE increases as the deviation parameter $k_{d}$ increases, which is in good consistency with our expectation. In Table 5, in combination with the third pose adjustment strategy in Ref. [45], the RSE is improved further, with a minimum of 11.76% for the improved sunflower and a maximum of 52.87% for the Dornier FIRST. It must be emphasized that, compared with the RSE adopting the regular panel and third pose adjustment strategy (i.e., $k_{d}=0$ in Table 5), the RSE adopting the deviation-angle panel in Table 4 can be higher if $k_{d}\geq 0.6$ .

In summary, adopting a deviation-angle panel is more efficient than pose adjustment for improving the RSE. If both approaches are combined, the RSE will be improved to the maximum extent. All of these facts demonstrate that the newly proposed segmentation scheme of the RSSA possesses significant superiority in improving the radial stowing efficiency.

## 5. Structure Design Based on Universal Joint Coupling Mechanism

Based on the configuration presented in Section 4.4, a concrete CAD model of a Cassegrain antenna is established. According to the resulting fixed point *P* and axial vector v , the precise position of the rotation axis can be determined, as shown in Table 2, based on which the process of structure design is demonstrated as follows.


**(1) Composite panel**
Although the reflective surface of the panel can be machined into an ideal paraboloid, its high accuracy cannot be guaranteed under harsh space environments without an elaborate panel design. In space, the environment temperature approximately ranges from 
$-100^{{}^{\circ}C}$
 to 
$200^{{}^{\circ}C}$
; hence, the heat-resistant material is indispensable for fabricating the panel. Also, high-strength and high-stiffness materials are needed to avoid possible resonance during launch, deployment and altitude maneuver. To meet these two requirements, the carbon-fiber-reinforced plastic (CFRP) sandwich structure is adopted. As shown in Figure 10b, the two skins are made of M55J CFRP, and the honeycomb core is made of T300 CFRP. The related research has reported that this kind of sandwich structure is capable of sustaining a high surface accuracy under severe on-orbit thermal environments [64,65,66].


**(2) Supporting framework**
To support the panels and further improve their strength and stiffness, a truss-type framework is also adopted, in which its constituent rods are made of T300 CFRP to reduce the weight, as shown in Figure 10c. In addition, diverse optimizations are performed with respect to the framework in terms of physical dimensions, position and topology to further lower its weight. Note that the fully deployed antenna is closely similar to the ground-based reflector antennas; thus, their topology optimization techniques [67,68,69] can be directly applied to this antenna by supplementing only one additional constraint. The constraint requires that the adjacent petals shall not interfere with each other during deployment, which was reported in Ref. [38]. (This optimization research will be presented in future studies.)


**(3) Deployment mechanism**
Typically, there are two ways to deploy the antenna: deploying all petals with one motor or deploying with the same number of motors as the petals. The first approach requires a synchronous device; however, its cost may not necessarily be lower than that of the second approach considering the expenditure. In addition, each petal is deployed by a single motor, which can reduce the position error of the petal to a large extent; therefore, the second approach is adopted. It is known that the universal joint coupling (UJC) is a compact structure capable of bearing high torque loading, which is appropriate to use for deploying the RRSAs due to their heavy weight and narrow space in placing deployment mechanisms. Figure 10a illustrates the deployment mechanism, from which it can be observed that each petal is mounted on the output rod of the UJC, and the input rod is connected through a hole with a reducer linked to the driving motor. The reducer is indispensable because of the extremely slow deployment process. According to the related reports, the deployment of Spektr-R takes one and a half hour, as shown in Figure 1. This operation can offer larger driving torque, which is of vital importance for large SSDAs due to their heavy petals. In this way, if all motors drive the input rods synchronously, the antenna deployment can be fulfilled without concerning the interference among petal deployment mechanisms.


**(4) Supporting and axis-adjusting device**
The EM performance of the antenna is influenced not only by the surface error caused by manufacturing but also by the position error largely induced by the rotation axis, which is equivalent to additional surface error. Therefore, strict rotation-axis accuracy must be guaranteed, to which a device capable of adjusting the axis position is introduced. A line in 3D space has five DOFs [70]; hence, the device must supply the output rod with at least five DOFs in order to precisely install the output rod. The ability of the input rod to move slightly on the hole plane provides two DOFs, and the telescopic function of the input rod provides an extra DOF, by which three translational DOFs are thus obtained. In addition, the universal joint provides the two other rotational DOFs, thereby verifying that adopting the UJC can guarantee the expected position of the output rod.


**(5) Base**
All the driving motors are anchored on the bottom plate of the base, and all axis-adjusting devices are mounted on the top plate, while the side of the base is hollowed out to realize the lightweight design. The bottom surface of the bottom plate base has bolt-fastening holes for mounting the antenna on the satellite platform, like Spektr-R shown in Figure 1.

The remaining work is to mount the petal on the output rod by sliding it on the rod until the resulting location makes the reflective surface of the petal exactly overlap with the ideal paraboloid. It can be observed from the animation simulation performed in *SolidWorks* that the antenna can be deployed successfully, to which three relevant screenshots of folded, intermediate and deployed states are, respectively, shown on the top side of Figure 10. We also observed that the whole deployment runs stably and smoothly, during which no interference is found, ensuring a uniform motion for the driving motors. All of the above facts suggest that the newly proposed RRSA can be successfully deployed in a stable and interference-free manner in a geometric and kinematic sense. In order to further confirm the effectiveness of the deployment of the RRSA, the deployment dynamics will be investigated in the subsequent section.

## 6. Trajectory-Planning-Based Deployment Dynamics

In order to further identify whether the proposed RRSA can be successfully deployed and to explore the dynamic characteristics of the deployment process, trajectory planning with an analytical method and software simulation is performed in this section.

### 6.1. Trajectory Planning

**Figure 11:**
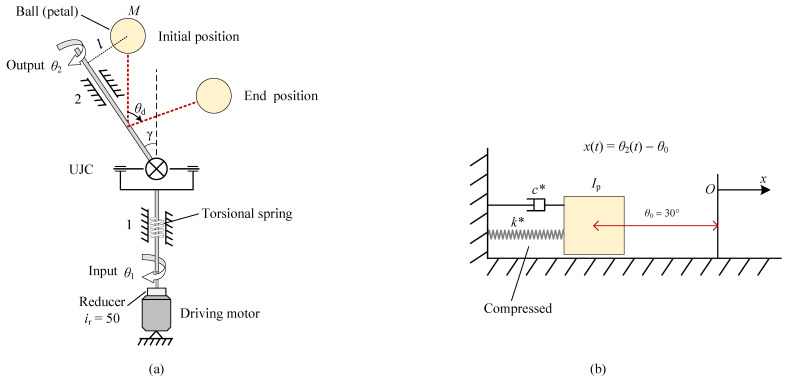
Dynamic model. (**a**) Equivalent dynamic model of petal deployment. (**b**) Simplified dynamic model of petal deployment. (1) Variable $\theta_{1}$ is the angular displacement of the input rod, variable $\theta_{2}$ is the angular displacement of the output rod; (2) the moment of inertia (MOI) of the ball is $I_{p}=I_{c}+Ml^{2}$ according to the parallel axis theorem, where $I_{c}$ is the MOI about the centroidal axis. After measuring in *SolidWorks*, we obtain that $I_{p}=0.2210618\;\mathrm{kg}\;m^{2}$ ; (3) the angle $\gamma =\langle v,{\left(0,0,-1\right)}^{T}\rangle =37.85^{{}^{\circ}}$ .

During deployment, the antenna will usually be deployed first by an angle with torsional springs to avoid dead points, and then deployed by motors [71]. This process can be approximated as the dynamic model shown in Figure 11a. It can be found from the figure that the petal is equivalent to a small ball anchored on the output rod with a mass *M* and distance *l* from the ball center to the output rod, and that the driving motor is connected with a reducer immediately linked to the input rod. In addition, a torsional spring is anchored at the input rod to provide the torque at the first deployment stage. Finally, assume that the reduction ratio is $i_{r}=50$ , the preloading angle of the spring is $\theta_{0}^{*}$ and its stiffness coefficient *k* and damping coefficient *c* are unknown parameters to be identified.

At the first deployment stage, i.e., the deployment with torsional springs, the time taken is assigned as $\left[0,t_{f_{1}}\right]$ . As for the second stage, i.e., the deployment with motors, the time taken is assumed to be within $\left[t_{f_{1}},t_{f_{2}}\right]$ , and the following values are taken: $t_{f_{1}}=60\;s$ , $t_{f_{2}}=180\;s$ and $t_{d}=t_{f_{2}}-t_{f_{1}}=120\;s$ . Furthermore, for the first stage, the additional boundary conditions

(18) $$\left\{\begin{array}{c} \theta_{2}\left(t_{f_{1}},k,c\right)=\theta_{0},\\ {\dot{\theta }}_{2}\left(t_{f_{1}},k,c\right)=v_{0}, \end{array}\right.$$

are supplemented, where $\theta_{0}=30^{{}^{\circ}}$ and $v_{0}=0.5^{{}^{\circ}}/s$ . Also, assume that, at $t=t_{f_{1}}$ , the spring is precisely in its equilibrium position.

Referring to Ref. [72], the Lagrangian function of the mechanical system shown in Figure 11a can be expressed as

(19) $$L=T-V=\frac{1}{2}I_{i}{\dot{\theta }}_{1}^{2}+\frac{1}{2}\left(I_{p}+I_{o}\right){\dot{\theta }}_{2}^{2}-\frac{1}{2}k{\left(\theta_{1}-\theta_{0}^{*}\right)}^{2},$$

where $I_{i}$ , $I_{p}$ and $I_{o}$ are the MOIs of the input rod, petal and output rod, respectively. Additionally, the dissipation function is

(20) $$Q=\frac{1}{2}c{\dot{\theta }}_{1}^{2}.$$

According to Ref. [73], we know that

(21) $$\theta_{1}=\arctan\left(\cos\gamma \tan\theta_{2}\right),$$

from which we can obtain that $\theta_{0}^{*}=24.51^{{}^{\circ}}$ . Substituting Equation (21) into Equations (20) and (19) yields a dissipation function $Q^{*}$ and Lagrangian function $L^{*}$ , both of which containing only variable $\theta_{2}$ . As a result, the dynamic equation with respect to $\theta_{2}$ is formulated as

(22) $$\frac{d}{dt}\frac{\partial L^{*}}{\partial {\dot{\theta }}_{2}}-\frac{\partial L^{*}}{\partial \theta_{2}}+\frac{\partial Q^{*}}{\partial {\dot{\theta }}_{2}}=0.$$

**Figure 12:**
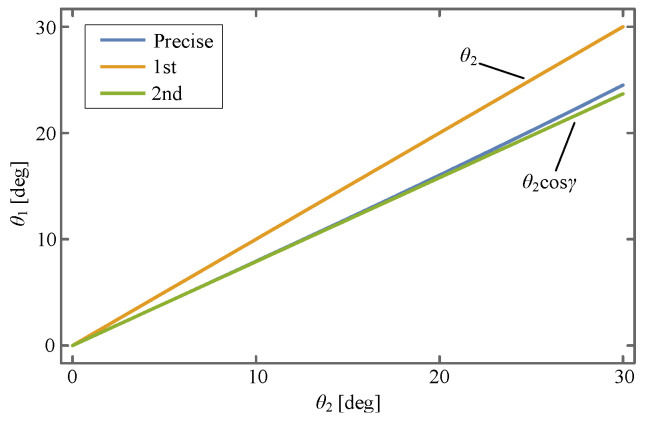
Linearization of $\theta_{2}$ . This figure compares two kinds of linearization methods for $\theta_{2}$ .

The explicit expression of Equation (22) is shown in Equation (A1), from which we know that it is a considerably complex nonlinear ordinary differential equation (ODE) and does not have an analytical solution. This causes difficulty in identifying the values of *k* and *c*, which can be attributed to the nonlinearity of $\theta_{1}$ . To solve this problem, it is of vital necessity to linearize $\theta_{1}$ . One reasonable method is to suppose that the velocities of the input and output rod remain approximately the same, i.e., let $\theta_{1}=\theta_{2}$ . As for another method, the expression in Equation (21) is expanded at $\theta_{2}=0$ using Taylor’s expansion as follows:

(23) $$\theta_{1}=\theta_{2}\cos\gamma +\frac{1}{3}\theta_{2}^{3}\left(\cos\gamma -\cos^{3}\gamma \right)+O\left(\theta_{2}^{4}\right),$$

where the second and third term are truncated, i.e., take $\theta_{1}=\theta_{2}\cos\gamma$ . To explore the approximation performance, these two methods are visualized in Figure 12, from which it is concluded that the second liberalization method exhibits a better approximation effect and thus is taken as the preferable method.

Meanwhile, the MOIs of the input and output rod are negligible because they are far lower than those of the petal, based on which the Lagrange function in Equation (19) is simplified to

(24) $$\left\{\begin{array}{c} L=\frac{1}{2}I_{p}{\dot{\theta }}_{2}^{2}-\frac{1}{2}k{\left(\cos\gamma \theta_{2}-\cos\gamma \theta_{0}\right)}^{2}=\frac{1}{2}I_{p}{\dot{\theta }}_{2}^{2}-\frac{1}{2}k^{*}{\left(\theta_{2}-\theta_{0}\right)}^{2},\\ Q=\frac{1}{2}\left(c\cos^{2}\gamma \right){\dot{\theta }}_{2}^{2}=\frac{1}{2}c^{*}{\dot{\theta }}_{2}^{2}, \end{array}\right.$$

where $k^{*}=k\cos^{2}\gamma$ and $c^{*}=c\cos^{2}\gamma$ . After conducting the above operations and according to Equation (24), the torsional spring can be considered to be mounted on the output rod, with a new stiffness coefficient $k^{*}$ and damping coefficient $c^{*}$ . The mechanical system in Figure 11a is essentially simplified into a mass–spring–damper system. For clearer illustration, the tension spring is utilized as an analogy to the torsional one, and eventually the corresponding simplified dynamic model is demonstrated in Figure 11b. According to Ref. [74] and Figure 11b, the dynamic equation of the first deployment stage can be formulated as

(25) $$\left\{\begin{array}{c} \ddot{x}\left(t\right)+2\zeta \omega_{n}\dot{x}\left(t\right)+\omega_{n}^{2}x\left(t\right)=0,\\ x\left(0\right)=-\theta_{0},\\ \dot{x}\left(0\right)=0, \end{array}\right.$$

where $\omega_{n}=\sqrt{k^{*}/I_{p}}$ , $\zeta =\frac{c^{*}}{2\sqrt{k^{*}/I_{p}}}$ and hence the circular frequency $\omega_{d}=\sqrt{1-\zeta^{2}}\omega_{n}$ . Also, based on the geometric relation demonstrated in Figure 11b, the angular displacement of the petal is

(26) $$\theta_{2}\left(t\right)=x\left(t\right)-\theta_{0}.$$

By referring to Ref. [74], the exact solution of $\theta_{2}$ can be obtained by simultaneously solving Equations (25) and (26) (see Equation (A2)), to which the solution is a function of *k* and *c*.

**Figure 13:**
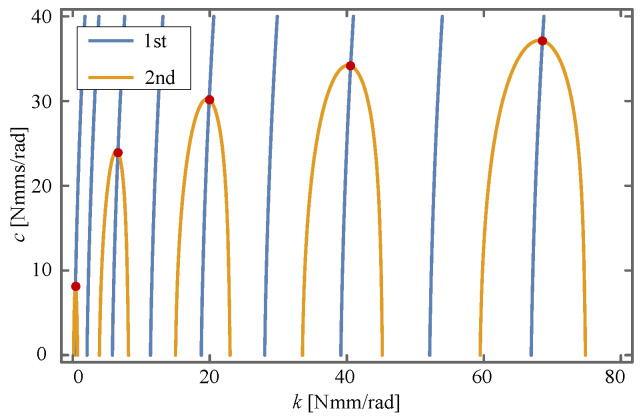
Points of intersection. The rough intersection locations are used as the initial values of Newton’s method.

By substituting the solution of $\theta_{2}$ into Equation (18), a system of equations (SOE) concerning *k* and *c* is thus obtained. This SOE is a nonlinear one and includes several trigonometric functions, so it may have multiple sets of solutions. For an intuitive insight into the distribution of these solutions, the SOE is drawn as implicit functions in Figure 13, where the red points represent the solutions. Therefore, these solutions can be obtained by taking the points’ rough locations as the iterative initial values and solving the SOE with Newton’s method, which is reported in Table 6.

**Figure 14:**
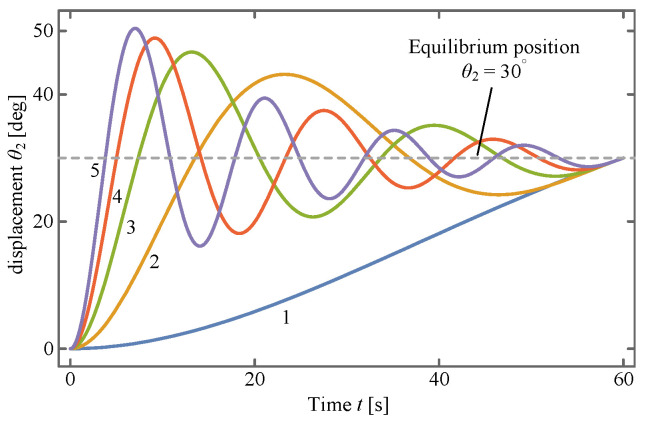
Behavior of solution. The first curve is the expected one.

Substituting these solutions back into Equation (A2) obtains the solutions of $\theta_{2}$ with different *k* and *c*, which are drawn in Figure 14. It can be found from the figure that all of the curves are oscillatory during the first deployment stage except for the first curve, amongst which each one of them appears as more noticeable than the next.

The above fact can also be concluded from Table 6, in which only the semi-period of the first solution is greater than 60 s. For other solutions, the semi-period becomes smaller, causing more intensive oscillation. In summary, only the first solution is the feasible one, denoted as $\theta_{2}^{\left(1\right)}\left(t\right)$ .

**Figure 15:**
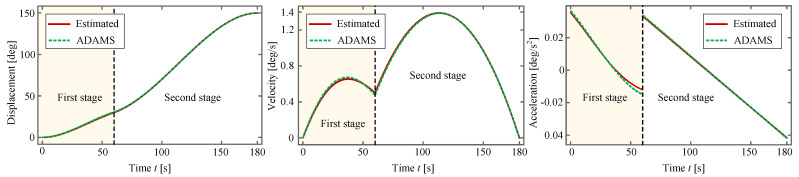
Planned motion. The displacement simulated by *ADAMS* is $\theta_{0}\left(t_{f_{1}}\right)=30.60^{{}^{\circ}}$ at $t=t_{f_{1}}$ , and the velocity is $v_{0}\left(t_{f_{1}}\right)=0.48^{{}^{\circ}}/s$ .

After the first deployment stage is accomplished, the action on the petal of the torsional springs is released, at which moment the driving motor’s torque is exerted to the petal immediately, thereby initiating the second deployment stage. In this phase, the planned trajectory of the petal is prescribed as a cubic curve, and the duration is $t_{d}=120\;s$ ; hence, its expression, denoted as $\theta_{2}^{\left(2\right)}\left(t\right)$ , can be written as

(27) $$\theta_{2}^{\left(2\right)}\left(t\right)=\theta_{0}+v_{0}t+\left[\frac{3}{t_{d}^{2}}\left(\theta_{d}-\theta_{0}\right)-\frac{2}{t_{d}}v_{0}\right]t^{2}+\left[-\frac{2}{t_{d}^{3}}\left(\theta_{d}-\theta_{0}\right)+\frac{1}{t_{d}^{2}}v_{0}\right]t^{3}\;0\leq t\leq t_{d},$$

according to Ref. [75]. As a result, during the entire deployment process, the expression of the planned trajectory can be written as

(28) $$\theta_{2}\left(t\right)=\left\{\begin{array}{c} \theta_{2}^{\left(1\right)}\left(t\right)\;0\leq t\leq t_{f_{1}},\\ \theta_{2}^{\left(2\right)}\left(t-t_{f_{1}}\right)\;t_{f_{1}}<t\leq t_{f_{2}}, \end{array}\right.$$

to which its motion curves, including displacement, velocity and acceleration, are shown in Figure 15. In the figure, the red line represents the theoretically estimated result from Equation (28), and the green dashed line represents the result obtained by the commercial software *Automatic Dynamic Analysis of Mechanical Systems (ADAMS)* employing the same *k* and *c*.

**Figure 16:**
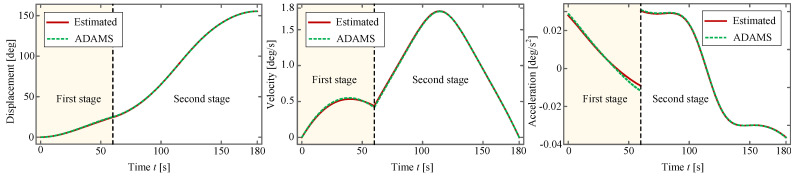
Input motion. (1) Required input motions of the torsional spring and the driving motor, respectively; (2) comparison between the two input motions obtained by theoretical estimation and by *ADAMS*.

According to Ref. [73], the correlation between the input angle $\theta_{1}$ and the output angle $\theta_{2}$ can be described by

(29) $$\theta_{1}\left(t\right)=\left\{\begin{array}{c} \arctan\left(\cos\gamma \tan\theta_{2}^{\left(2\right)}\right)\;0<t\leq t_{90},\\ \pi +\arctan\left(\cos\gamma \tan\theta_{2}^{\left(2\right)}\right)\;t_{90}<t\leq t_{f_{2}}, \end{array}\right.$$

where $t_{90}$ is the time when $\theta_{1}=90^{{}^{\circ}}$ . This expression along with its first-order and second-order derivatives, as well as the simulated results obtained by *ADAMS*, is drawn in Figure 16.

It can be observed from Figure 15 and Figure 16 that these two kinds of curves, i.e., the theoretically estimated one and the one obtained by *ADAMS*, almost overlap with each other, which verifies the effectiveness of the planned deployment. Owing to this fact, it suffices for us to rely solely on the theoretical method to conduct trajectory planning of the antenna. Considering the fact that the displacement and the velocity are continuous whereas the acceleration is not, soft shock occurs at the moment when the torsional-spring-driven deployment switches to the motor-driven one. Fortunately, the soft shock does not impose a perceptible influence on the whole system because the deployment process is considerably slow.

### 6.2. Driving Torque

The effectiveness in kinematics is based on the assumption that the antenna can be successfully deployed by torsional springs and driving motors. This requires that the effectiveness in kinetics shall be confirmed and validated; in other words, the driving force or the torque must be proven as reasonable. At the first deployment stage, for each petal, we have

(30) $$T_{i}^{\left(1\right)}\left(t\right)=k\left(\theta_{0}^{*}-\theta_{1}\right)=k\cos\gamma \left(\theta_{0}-\theta_{2}\right)\;0\leq t\leq t_{f_{1}}.$$

where $T_{i}^{\left(1\right)}$ denotes the torque of each torsional spring at the first deployment stage. On the other hand, at the second stage, following the kinetic energy theorem for each petal, we arrive at

(31) $$\int_{t_{f_{1}}}^{t}T_{i}^{\left(2\right)}{\dot{\theta }}_{1}dt=\frac{1}{2}I_{p}{\dot{\theta }}_{2}^{2}-\frac{1}{2}I_{p}v_{0}^{2}=\frac{1}{2}I_{p}\left({\dot{\theta }}_{2}^{2}-v_{0}^{2}\right)\;t_{f_{1}}\leq t\leq t_{f_{2}},$$

if only considering the MOI of the petal and neglecting all the frictions. Thereinto, $T_{i}^{\left(2\right)}$ denotes the torque exerted on the input rod at the second deployment stage. By differentiating both sides of Equation (31), we have

(32) $$T_{i}^{\left(2\right)}{\dot{\theta }}_{1}=I_{p}{\dot{\theta }}_{2}{\ddot{\theta }}_{2}.$$

Hence, the torque of the input rod is

(33) $$T_{i}^{\left(2\right)}\left(t\right)=\frac{I_{p}{\dot{\theta }}_{2}{\ddot{\theta }}_{2}}{{\dot{\theta }}_{1}}=\frac{I_{p}{\dot{\theta }}_{2}{\ddot{\theta }}_{2}}{{\dot{\theta }}_{2}\cos\gamma }=\frac{I_{p}{\ddot{\theta }}_{2}}{\cos\gamma }\;t_{f_{1}}\leq t\leq t_{f_{2}},$$

and the total estimated driving torque throughout the deployment process can be expressed as

(34) $$T_{i}\left(t\right)=\left\{\begin{array}{c} k\cos\gamma \left(\theta_{0}-\theta_{2}\right)\;0\leq t\leq t_{f_{1}},\\ \frac{I_{p}{\ddot{\theta }}_{2}}{\cos\gamma }\;t_{f_{1}}<t\leq t_{f_{2}}. \end{array}\right.$$

Consequently, the driving torque of the driving motor is

(35) $$T_{m}\left(t\right)=\frac{I_{p}{\ddot{\theta }}_{2}}{i_{r}\cos\gamma }\;t_{f_{1}}\leq t\leq t_{f_{2}}$$

**Figure 17:**
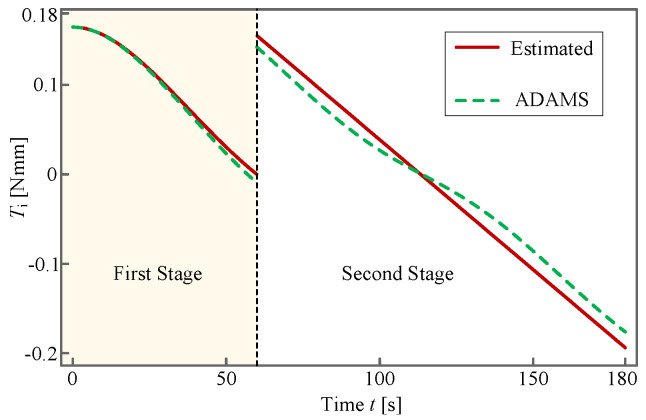
Input torque. Torques of the UJC’s input rod obtained by theoretical estimation and *ADAMS*, respectively.

**Figure 18:**
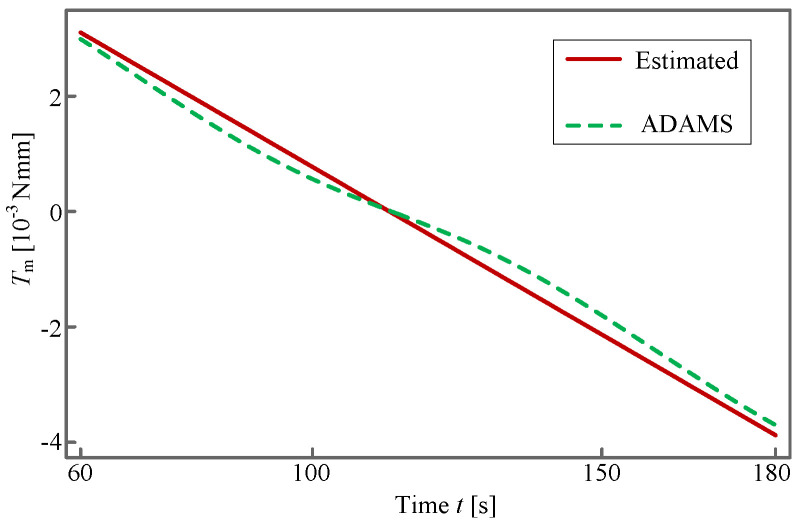
Driving torque. When simulating using *ADAMS*, the component MOI of the reducer is considered.

By differentiating Equation (28) and substituting the result into Equations (34) and (35), the explicit expressions of $T_{i}$ and $T_{m}$ can be obtained. However, these explicit expressions are not presented here due to their complexity. Ultimately, the theoretically estimated result and *ADAMS*-simulated one are presented in Figure 17 and Figure 18.

It can be observed from Figure 17 and Figure 18 that the torques of the input rod and of the driving motor obtained by theoretical estimation and *ADAMS*, respectively, are in good consistency with each other, thereby verifying the correctness of our trajectory planning. In addition, the varying tendency of the corresponding curves is similar to that in Figure 15c. This phenomenon is in agreement with our expectation since the torque is proportional to the angular acceleration, to which the petal contributes most of the MOI. According to Figure 18, the maximum torque is approximately $3\times 10^{-3}\;\mathrm{Nmm}$ and the minimum is about $-4\times 10^{-3}\;\mathrm{Nmm}$ , both of which staying within a reasonable range of magnitude for deploying a 0.2 kg petal with an MOI of $0.22\;\mathrm{kg}\;m^{2}$ . Based on the results in Figure 15, Figure 16, Figure 17 and Figure 18, we argue that the antenna can be successfully deployed and exhibits excellent dynamical quality.

Based on the research in this paper, our future work will focus on prototype fabricating, experiments of deployment dynamics and deployment control, etc., to verify the effectiveness of the proposed RRSA further.

## 7. Conclusions

This study proposed a new kind of RRSA by deviating the radial segmentation line by an angle, which utilized most of the advantages of the regular-panel antenna described in Section 2.4. By adopting this new panel, the compact folded pose was derived to achieve high RSE. The deployment scheme is based on Euler’s rotation theorem, and adopts rigid-body registration algorithms to obtain the transformation matrix. Inspired by the idea of parameter identification in robotics, the PECM was established in order to satisfy the prescribed geometric constraints, to which the CPI algorithm was developed for the antenna configuration with high RSE. Extensive simulations have verified the effectiveness of the algorithm.

Then, the RSEs obtained by the CPI algorithm were compared with those of the existing RRSAs. The results suggest that the RSE has improved markedly, and that adopting the deviation-angle panel is more efficient than conducting pose adjustment in terms of achieving higher RSE. Alternatively, the RSE can be further improved by adopting a combination of these two approaches.

Furthermore, based on the obtained configuration and rotation axis, the process of mechanism/structure design was performed. The entire deployment system mainly consists of five parts, i.e., a composite panel, supporting framework, UJC deployment mechanism, axis-adjusting device and base. The CAD model of the antenna was established by *SolidWorks*, and the kinematic simulation revealed that the antenna deployment is stable and interference-free in a geometric and kinematic sense.

Eventually, we performed deployment dynamics based on trajectory planning to further confirm that the antenna can be successfully deployed and to explore the dynamic characteristics of the antenna. Our designed mechanical system is equivalent to a UJC attached with a small ball on the output rod and a torsional spring and a driving motor on the input rod. The system was then simplified into a mass–spring–damper system using a linearization technique, after which the oscillation-free mechanical parameters were immediately identified for the spring-driven stage and a cubic trajectory was planned for the motor-driven stage. The theoretically estimated result and the *ADAMS*-simulated one are in good consistency with each other, suggesting that the antenna can be deployed successfully and stably in a dynamical sense.

To conclude, the proposed RRSA in this study is capable of supplying higher radial stowing efficiency. Kinematic simulation combined with analytical and simulated dynamics demonstrates that the deployment process is stable and interference-free. The above facts suggest that our proposed RRSA provides meaningful references in the field of rigid-reflector spaceborne antennas with excellent dynamical quality and higher radial stowing efficiency.

## Figures and Tables

**Table 1:** Characteristics of antenna concepts.

Antenna Concept	Specification						
	Number of Units n	Panel Number of Each Unit m	Supporting Structure	Deployment Mechanism	Surface Accuracy	Symmetry	Number of Molds
Sunflower	6	3	No	Complex	Medium	Medium	3
SSDA	6	3	No	Complex	Medium	Medium	4
Regular panel	18	1	Yes	Simple	High	High	2
Deviation-angle panel	18	1	Yes	Simple	High	High	2

The molds contain the one for manufacturing the central hub.

**Table 2:** Structural parameters and results.

Structure		Solution	
Parameter	Value	Parameter	Value
Initial aperture $D_{a}$ (mm)	2000	Theoretical and simulated maximum aperture $\left(D_{a}^{t},D_{a}^{*}\right)$ (mm)	(2190.726, 2190.726)
Focus/diameter ratio η	0.5	Transformation matrix $T^{*}$	$\left[\begin{array}{cccc} -0.460911 & -0.742475 & 0.486099 & 516.74\\ 0.0482902 & -0.567922 & -0.821664 & 289.684\\ 0.886132 & -0.35524 & 0.297616 & -158.249\\ 0 & 0 & 0 & 1 \end{array}\right]$
Number of panels *n*	18	Compensation parameters $\left(k_{1}^{*},k_{2}^{*}\right)$	(0.118094,0.861829)
Fairing diameter *s* (mm)	5	Compensation parameter $\beta^{*}$ (deg)	37.413
Vertical displacement *h* (mm)	10	Fixed point *P*	${\left(274.239,176.829,31.2453,1\right)}^{T}$
Fairing diameter $D_{f}$ (mm)	740	Theoretical and simulated RSE $\left(\zeta_{t},\zeta_{s}\right)$	(2.960, 2.960)
Initial RSE $\zeta_{0}$	2.703	Deployment angle $\theta_{d}$ (deg)	149.952
Deviation parameter $k_{d}$	0.6	Axial vector v	${\left(-0.465753,0.399456,-0.789626\right)}^{T}$
–	–	Residual $E^{*}$	$3.483\times 10^{-13}$

**Table 3:** Structural parameters of existing RRSAs.

Parameter	Antenna								
	Dornier FIRST [31]	Dornier MEA	RadioAstron	MilliMetron	NPSSDA [32]	Uniaxial Model [36]	CFRP Model [40]	Sunflower	Improved Sunflower
Aperture									
diameter (m)	8.0	4.7	10.0	10.0	1.2	1.2	1.0	4.42	1.5
Focal length (m)	[2.4]	[1.41]	4.30	3.00	0.48	0.48	0.37	1.77	1.25
Panel number	24	24	27	24	24	20	30	18	18
RSE	2.78	2.78	3.33	3.33	3.16	2.82	3.76	2.29	2.70

[·] represents that its value is unknown from existing literature, so its focus/diameter ratio takes 0.3 by default.

**Table 4:** Comparison of RSE I.

Antenna	Deviation Parameter $k_{d}$											Improvement Rate (%)	
	**0**	0.1	0.2	0.3	0.4	0.5	0.6	0.7	0.8	0.9	1.0	$\eta_{\min}$	$\eta_{\max}$
Dornier FIRST	3.86	3.87	3.88	3.90	3.92	3.94	3.96	3.98	4.01	4.04	4.08	39.15	46.70
Dornier MEA	3.86	3.87	3.88	3.90	3.92	3.94	3.96	3.98	4.01	4.04	4.08	39.15	46.70
RadioAstron	4.33	4.34	4.36	4.37	4.39	4.41	4.43	4.46	4.48	4.51	4.55	30.45	36.50
MilliMetron	3.86	3.87	3.88	3.90	3.92	3.94	3.96	3.98	4.01	4.04	4.08	16.17	22.47
NPSSDA	3.86	3.87	3.89	3.90	3.92	3.94	3.97	4.00	4.03	4.06	4.09	22.51	29.54
Uniaxial Model	3.23	3.25	3.26	3.28	3.31	3.33	3.36	3.39	3.43	3.47	3.51	15.10	24.47
CFRP Model	4.81	4.82	4.83	4.84	4.86	4.87	4.89	4.91	4.94	4.96	4.99	28.11	32.78
Sunflower	2.92	2.93	2.95	2.98	3.00	3.03	3.06	3.10	3.14	3.18	3.23	28.10	40.86
Improved Sunflower	2.92	2.94	2.96	2.99	3.02	3.05	3.08	3.12	3.16	3.20	3.25	8.93	20.37

(1) Parameters $\eta_{\min}$ and $\eta_{\max}$ are the minimum and maximum improvement rate relative to the original RSE in Table 3, respectively, when the deviation parameter $k_{d}$ ranges from 0.1 to 1.0 in a step of 0.1; (2) for each antenna in Table 3, the value of interference parameter *s* takes 0 for comparison with the RSE in Ref. [45]; (3) the second column ( $k_{d}=0$ ) colored in light blue denotes that the panel is the regular panel in Section 2.4; (4) the same situation applies to the table below.

**Table 5:** Comparison of RSE II.

Antenna	Deviation Parameter $k_{d}$											Improvement Rate (%)	
	**0**	0.1	0.2	0.3	0.4	0.5	0.6	0.7	0.8	0.9	1.0	$\eta_{\min}$	$\eta_{\max}$
Dornier FIRST	3.96	3.97	3.99	4.01	4.03	4.06	4.09	4.12	4.16	4.20	4.25	42.80	52.87
Dornier MEA	3.96	3.98	3.99	4.01	4.03	4.06	4.09	4.12	4.15	4.19	4.25	43.08	52.81
RadioAstron	4.41	4.42	4.44	4.46	4.48	4.51	4.52	4.56	4.58	4.62	4.66	32.82	39.87
MilliMetron	3.96	3.98	3.99	4.01	4.04	4.06	4.09	4.12	4.16	4.20	4.25	19.50	27.62
NPSSDA	3.94	3.95	3.97	4.00	4.02	4.05	4.07	4.11	4.14	4.18	4.23	25.13	33.92
Uniaxial Model	3.34	3.35	3.37	3.40	3.43	3.46	3.49	3.52	3.56	3.61	3.67	18.92	30.23
CFRP Model	4.87	4.86	4.88	4.90	4.92	4.95	4.97	5.00	5.03	5.06	5.10	29.29	35.62
Sunflower	3.04	3.06	3.08	3.10	3.13	3.17	3.20	3.23	3.28	3.33	3.40	33.46	48.33
Improved Sunflower	3.00	3.02	3.04	3.07	3.09	3.13	3.16	3.20	3.25	3.30	3.35	11.76	24.20

**Table 6:** Solution of *k* and *c*.

No.	k[Nmm/rad]	c[Nmms/rad]	Semi-Period $\pi /\omega_{d}\left[s\right]$
1	0.398	8.139	97.703
2	6.596	23.943	23.241
3	19.921	30.166	13.161
4	40.555	34.168	9.165
5	68.545	37.123	7.027

## Data Availability

Data are contained within the article.

## Appendix A. Detailed Explanation of Completeness, Continuity and Minimality of PECM

Firstly, unknowns $\left\{z_{A_{1}},r_{B_{1}},z_{B_{1}}\right\}$ can be obtained by solving Equation (10) if given the values of $k_{1}$ , $k_{2}$ and β ; then, the coordinates of point set ${\left\{A_{1},B_{1},P\right\}}^{\prime }$ is obtained, which means that the pose of the folded panel is known. Hence, compensation parameters $k_{1}$ , $k_{2}$ and β can completely characterize the folded panel pose; that is, the PECM has completeness.

Secondly, according to Equations (8) and (9), all expressions in Equation (10) are elementary functions containing $k_{1}$ , $k_{2}$ and β . Thus, unknowns $\left\{z_{A_{1}},r_{B_{1}},z_{B_{1}}\right\}$ are implicit continuous functions about $k_{1}$ , $k_{2}$ and β , which are determined by Equation (10). This fact indicates that the folded panel pose is a continuous function with respect to $k_{1}$ , $k_{2}$ and β . As a result, the continuity of the PECM has been proven.

Thirdly, according to Equation (9), the folded panel pose ( ${\left\{A_{1},B_{1},P\right\}}^{\prime }$ ) is determined by $k_{1}$ , $k_{2}$ and β . If the value of β is not given, $r_{A_{1}}^{\prime }$ and $r_{B_{1}}^{\prime }$ will be unknown. If the value of $k_{1}$ or $k_{2}$ is not given, $r_{P}^{\prime }$ will be unknown. All of the above facts show that $k_{1}$ , $k_{2}$ and β are the minimum parameter numbers used to determine the folded panel pose, thereby verifying suggestions that the PECM has minimality.

## Appendix B. Dynamic Equation of Deployment Mechanism

The dynamic equation of the mechanical system in Figure 11a during the first deployment stage:

(A1)
$$\begin{array}{c} \left[I_{p}+I_{o}+\frac{I_{i}\cos^{2}\gamma \sec^{4}\theta_{2}}{{\left(\cos^{2}\gamma \tan^{2}\theta_{2}+1\right)}^{2}}\right]{\ddot{\theta }}_{2}+\frac{2I_{i}\sin^{2}\gamma \cos^{2}\gamma \tan\theta_{2}\sec^{4}\theta_{2}}{{\left(\cos^{2}\gamma \tan^{2}\theta_{2}+1\right)}^{3}}{\dot{\theta }}_{2}^{2}+\frac{c\cos^{2}\gamma \sec^{4}\theta_{2}}{{\left(\cos^{2}\gamma \tan^{2}\theta_{2}+1\right)}^{2}}{\dot{\theta }}_{2}+\\ \frac{k\cos\gamma \sec^{2}\theta_{2}\left[\arctan\left(\cos\gamma \tan\theta_{2}\right)-\theta_{0}^{*}\right]}{\cos^{2}\gamma \tan^{2}\theta_{2}+1}=0, \end{array}$$

where $\theta_{0}^{*}=\arctan\left(\cos\gamma \tan\theta_{0}\right)$ .

The exact solution of Equation (25):

(A2)
$$\theta_{2}\left(t\right)=\theta_{0}\left[-\frac{\zeta e^{-\zeta \omega_{n}t}\sinh\left(\sqrt{\zeta^{2}-1}\omega_{n}t\right)}{\sqrt{\zeta^{2}-1}}-e^{-\zeta \omega_{n}t}\cosh\left(\sqrt{\zeta^{2}-1}\omega_{n}t\right)+1\right],$$

where $\omega_{n}=\sqrt{k^{*}/I_{p}}$ , $\zeta =\frac{c^{*}}{2\sqrt{k^{*}/I_{p}}}$ , $k^{*}=k\cos^{2}\gamma$ and $c^{*}=c\cos^{2}\gamma$ .

## References

[1] Zhang Y., Ru W., Yang G. (2016). Deployment analysis considering the cable-net tension effect for deployable antennas. Aerosp. Sci. Technol..

[2] Zhang Y., Li N., Yang G. (2017). Dynamic analysis of the deployment for mesh reflector deployable antennas with the cable-net structure. Acta Astronaut..

[3] Li B., Qi X., Huang H. (2016). Modeling and analysis of deployment dynamics for a novel ring mechanism. Acta Astronaut..

[4] Nie R., He B., Hodges D. (2019). Integrated form finding method for mesh reflector antennas considering the flexible truss and hinges. Aerosp. Sci. Technol..

[5] Han B., Xu Y., Yao J. (2019). Design and analysis of a scissors double-ring truss deployable mechanism for space antennas. Aerosp. Sci. Technol..

[6] Yuan P., He B., Zhang L. (2021). Pretension modeling and form-finding for cable-network antennas with varying topologies and parameters. Aerosp. Sci. Technol..

[7] Zhang J., He B., Zhang L. (2021). High surface accuracy and pretension design for mesh antennas based on dynamic relaxation method. Int. J. Mech. Sci..

[8] Sun Z., Yang D., Duan B. (2021). Structural design, dynamic analysis, and verification test of a novel double-ring deployable truss for mesh antennas. Mech. Mach. Theory.

[9] He B., Li K., Nie R. (2022). Deployment modeling for soft cable networks from slack to tension. Int. J. Mech. Sci..

[10] Thomson M. The Astromesh deployable reflector. Proceedings of the IEEE Antennas and Propagation Society International Symposium. 1999 Digest. Held in Conjunction with: USNC/URSI National Radio Science Meeting (Cat. No. 99CH37010).

[11] Wang Z., Li T., Cao Y. (2013). Active shape adjustment of cable net structures with PZT actuators. Aerosp. Sci. Technol..

[12] Du J., Zong Y., Bao H. (2013). Shape adjustment of cable mesh antennas using sequential quadratic programming. Aerosp. Sci. Technol..

[13] Du J., Bao H., Cui C. (2014). Shape adjustment of cable mesh reflector antennas considering modeling uncertainties. Acta Astronaut..

[14] Zhang Y., Dong B., Yang G. (2020). Design technique for a shaped-reflector antenna with a three-layer cable net structure. IEEE Trans. Antennas Propag..

[15] Du J., Zhang Y., Wang C. (2021). Robust optimal design for surface accuracy of mesh reflectors considering cable length inaccuracy. J. Aerosp. Eng..

[16] Sun Z., Zhang Y., Yang D. (2021). Structural design, analysis, and experimental verification of an H-style deployable mechanism for large space-borne mesh antennas. Acta Astronaut..

[17] Sauder J., Chahat N., Hodges R. Designing, building, and testing a mesh Ka-band parabolic deployable antenna (KaPDA) for CubeSats. Proceedings of the 54th AIAA Aerospace Sciences Meeting.

[18] Manohar V., Kovitz J., Rahmat-Samii Y. Ka band umbrella reflectors for CubeSats: Revisiting optimal feed location and gain loss. Proceedings of the 2016 International Conference on Electromagnetics in Advanced Applications (ICEAA).

[19] Wang P., Wang F., Shi T. (2017). Thermal distortion compensation of a high precision umbrella antenna. Proceedings of the Journal of Physics: Conference Series.

[20] Tang Y., Shi Z., Li T. (2019). Double-layer cable-net structures for deployable umbrella reflectors. J. Aerosp. Eng..

[21] Yang D., Liu J., Zhang Y. (2017). Optimal surface profile design of deployable mesh reflectors via a force density strategy. Acta Astronaut..

[22] Yang D., Zhang Y., Li P. (2018). Numerical form-finding method for large mesh reflectors with elastic rim trusses. Acta Astronaut..

[23] Tang Y., Li T., Liu Y. (2019). Minimization of cable-net reflector shape error by machine learning. J. Spacecr. Rocket..

[24] Zhang J., Zhang Y., He Y. (2023). Active adjustment of space-borne cable-net antenna via a two-way shape memory alloy spring. Smart Mater. Struct..

[25] Ruze J. (1966). Antenna tolerance theory—A review. Proc. IEEE.

[26] Rudnitskiy A., Graauw T., Andrianov A. Millimetron Space Observatory. Proceedings of the 43rd COSPAR Scientific Assembly.

[27] (2023). Astro Space Center of Lebedev Physical Institute, Russian Academy of Sciences. https://www.millimetron.ru/en/general/antenna.

[28] Archer J. (1979). Advanced sunflower antenna concept development. Nasa Langley Res. Cent. Large Space Syst. Technol..

[29] Guest S., Pellegrino S. (1996). A new concept for solid surface deployable antennas. Acta Astronaut..

[30] Zeng X., Zhou Y., Yuan W. (2021). Optimal Design of Solid Surface Deployable Antennas Based on Twin-Bennett Linkage. China Mech. Eng..

[31] Dornier (1987). First Technology Study: Multisurface Control Mechanism for a Deployable Antenna.

[32] Huang H., Guan F., Pan L. (2018). Design and deploying study of a new petal-type deployable solid surface antenna. Acta Astronaut..

[33] Specht P. (1990). Dynamics of Large Reflectors: DAISY/MEA Reflector-Description. Dornier Technical Report.

[34] Kardashev N., Khartov V., Abramov V. (2013). “RadioAstron”—A telescope with a size of 300,000 km: Main parameters and first observational results. Astron. Rep..

[35] Schwarz S., Barho R. Deployment mechanisms for a 5 m unfurlable reflector. Proceedings of the European Space Mechanisms and Tribology Symposium.

[36] Huang H., Cheng Q., Zheng L. (2021). Development for petal-type deployable solid-surface reflector by uniaxial rotation mechanism. Acta Astronaut..

[37] Tan G., Duan X., Yang D. (2022). Parametric design optimization approach to petal-type solid surface deployable reflectors. Acta Astronaut..

[38] Tan G., Duan X., Niu D. (2022). Visual synthesis of uniaxial synchronous deployment mechanisms for solid-surface deployable antennas. Mech. Mach. Theory.

[39] Sayapin S., Shkapov P. (2016). Kinematics of deployment of petal-type large space antenna reflectors with axisymmetric petal packaging. J. Mach. Manuf. Reliab..

[40] Chae S., Oh Y., Lee S. (2018). Design and test of a deployment mechanism for the composite reflector antenna. J. Aerosp. Syst. Eng..

[41] (2023). Astro Space Center of Lebedev Physical Institute, Russian Academy of Sciences. http://www.asc.rssi.ru/radioastron/index.html.

[42] Kovalev Y., Kardashev N., Kellermann K. The RadioAstron space VLBI project. Proceedings of the 2014 XXXIth URSI General Assembly and Scientific Symposium (URSI GASS).

[43] Arkhipov M., Savel’ev V., Smirnov A. (2020). Solving the problem of the deployment kinematics of a large petal reflector. J. Mach. Manuf. Reliab..

[44] Elsayed E. (2021). Reliability Engineering.

[45] Tan G., Duan X., Ma J. (2022). Investigation of compact packing strategy for large rigid-panel deployable antennas. Mech. Mach. Theory.

[46] Guang C., Yang Y. (2018). An approach to designing deployable mechanisms based on rigid modified origami flashers. J. Mech. Des..

[47] The Xinhua News Agency, 15 September 2023. http://www.chinanews.com/gn/2020/05-06/9176071.shtml.

[48] Guo H., Li Z., Liu R. Parametric analysis and optimization and structure design of deployable solid-surface antenna. Proceedings of the Chinese Society of Space Research Symposium.

[49] Guo H., Li Z., Liu R. (2016). Twin–Bennett Linkage and One Type of Its Mobile Assemblies. Advances in Reconfigurable Mechanisms and Robots II.

[50] Chen Y., You Z. (2008). An Extended Myard Linkage and its Derived 6R Linkage. J. Mech. Des..

[51] Wang Z. (2020). Structure Design and Analysis of Solid Surface Antenna Based on Rigid Origami. Master’s Thesis.

[52] Feng H., Peng R., Zang S. (2020). Rigid foldability and mountain-valley crease assignments of square-twist origami pattern. Mech. Mach. Theory.

[53] Luo A., Liu H., Li Y. (2012). Structural Analysis of Flowerlike Deployable Antenna. China Mech. Eng..

[54] Blitzer R. (2022). Trigonometry.

[55] Kelley C. (2003). Solving Nonlinear Equations with Newton’s Method.

[56] Palais B., Palais R. (2007). Euler’s fixed point theorem: The axis of a rotation. J. Fixed Point Theory Appl..

[57] Palais B., Palais R., Rodi S. (2009). A Disorienting Look at Euler’s Theorem on the Axis of a Rotation. Am. Math. Mon..

[58] Wu J., Wang J., You Z. (2010). An overview of dynamic parameter identification of robots. Robot. Comput.-Integr. Manuf..

[59] Li Z., Li S., Luo X. (2021). An overview of calibration technology of industrial robots. IEEE/CAA J. Autom. Sin..

[60] Schröer K., Albright S., Grethlein M. (1997). Complete, minimal and model-continuous kinematic models for robot calibration. Robot. Comput.-Integr. Manuf..

[61] Gatti G., Danieli G. (2007). A practical approach to compensate for geometric errors in measuring arms: Application to a six-degree-of-freedom kinematic structure. Meas. Sci. Technol..

[62] Arun K., Huang T., Blostein S. (1987). Least-squares fitting of two 3-D point sets. IEEE Trans. Pattern Anal. Mach. Intell..

[63] Besl P., McKay N. (1992). Method for registration of 3-D shapes. Proceedings of the Sensor fusion IV: Control Paradigms and Data Structures.

[64] Qian Y., Lou Z., Hao X. Initial development of high-accuracy CFRP panel for DATE5 antenna. Proceedings of the Advances in Optical and Mechanical Technologies for Telescopes and Instrumentation II.

[65] Qian Y., Hao X., Shi Y. (2019). Deformation behavior of high accuracy carbon fiber-reinforced plastics sandwiched panels at low temperature. J. Astron. Telesc. Instruments Syst..

[66] Wei X., Li D., Xiong J. (2019). Fabrication and mechanical behaviors of an all-composite sandwich structure with a hexagon honeycomb core based on the tailor-folding approach. Compos. Sci. Technol..

[67] Leng G., Duan B. (2012). Topology optimization of planar truss structures with continuous element intersection and node stability constraints. Proc. Inst. Mech. Eng. Part J. Mech. Eng. Sci..

[68] Feng S., Duan B., Wang C. (2018). Topology optimization of pretensioned reflector antennas with unified cable-bar model. Acta Astronaut..

[69] Zhang S., Song J. (2021). Surface segmentation design using a weighting level set topology optimization method for large radio telescope antennas. Struct. Multidiscip. Optim..

[70] Saliklis E. (2019). Structures: A Geometric Approach.

[71] Li T. (2012). Deployment analysis and control of deployable space antenna. Aerosp. Sci. Technol..

[72] Goldstein H. (2011). Classical Mechanics.

[73] Wittenburg J. (2016). Shaft Couplings. Kinematics.

[74] Krodkiewski J. (2008). Mechanical Vibration.

[75] Craig J. (2018). Introduction to Robotics: Mechanics and Control.

